# A metal–organic framework for efficient water-based ultra-low-temperature-driven cooling

**DOI:** 10.1038/s41467-019-10960-0

**Published:** 2019-07-09

**Authors:** Dirk Lenzen, Jingjing Zhao, Sebastian-Johannes Ernst, Mohammad Wahiduzzaman, A. Ken Inge, Dominik Fröhlich, Hongyi Xu, Hans-Jörg Bart, Christoph Janiak, Stefan Henninger, Guillaume Maurin, Xiaodong Zou, Norbert Stock

**Affiliations:** 1 Institut für Anorganische Chemie, Christian-Albrechts-Universität Kiel, Max-Eyth-Str. 2, 24118 Kiel, Germany; 2 Department of Materials and Environmental Chemistry, Stockholm University, SE-106 91 Stockholm, Sweden; 3 Department Heating and Cooling Technologies, Group Sorption Materials, Fraunhofer-Institut für Solare Energiesysteme ISE, Heidenhofstrasse 2, 79110 Freiburg, Germany; 4 Institut Charles Gerhardt Montpellier, Université Montpellier, UMR 5253 CNRS ENSCM UM, 34095 Montpellier, France; 5 TU Kaiserslautern, Chair of Separation Science and Technology, P.O. Box 3049, 67653 Kaiserslautern, Germany; 6 Institut für Anorganische Chemie und Strukturchemie I, Heinrich-Heine-Universität Düsseldorf, Universitätsstraße 1, 40225 Düsseldorf, Germany

**Keywords:** Metal-organic frameworks, Porous materials, Energy

## Abstract

Efficient use of energy for cooling applications is a very important and challenging field in science. Ultra-low temperature actuated (*T*_driving_ < 80 °C) adsorption-driven chillers (ADCs) with water as the cooling agent are one environmentally benign option. The nanoscale metal-organic framework [Al(OH)(C_6_H_2_O_4_S)] denoted CAU-23 was discovered that possess favorable properties, including water adsorption capacity of 0.37 *g*_H2O_/*g*_sorbent_ around *p*/*p*_0_ = 0.3 and cycling stability of at least 5000 cycles. Most importantly the material has a driving temperature down to 60 °C, which allows for the exploitation of yet mostly unused temperature sources and a more efficient use of energy. These exceptional properties are due to its unique crystal structure, which was unequivocally elucidated by single crystal electron diffraction. Monte Carlo simulations were performed to reveal the water adsorption mechanism at the atomic level. With its green synthesis, CAU-23 is an ideal material to realize ultra-low temperature driven ADC devices.

## Abstract (web summary)

Ultra-low-temperature-actuated adsorption-driven chillers that rely on water as the cooling agent are desirable for energy-efficient and environmentally benign cooling devices. Here the authors show that the metal–organic framework of composition [Al(OH)(C_6_H_2_O_4_S)] displays high water uptake capacity, high stability, and driving temperatures as low as 60 °C.

## Introduction

Cooling devices, such as air conditioning (AC) units, are expected to become one of the largest contributors to global energy consumption. According to the International Energy Agency the use of air conditioners and electric fans currently accounts for 10% of global energy consumption^1^. The number of AC units is expected to triple over the next 30 years, particularly due to increased income in developing countries, many of which are located in tropical or subtropical regions of the world^2–4^. These widely used devices mostly consist of compressors using partially hydrogenated chlorofluorocarbons. These materials are typically flammable, toxic, or harmful to the environment and facing a fade out process by international regulations^5,6^.

To address the problem of hazardous, energy-intensive cooling devices, new energy efficient and green approaches must be developed. Although adsorption driven chillers have been used for over a century, they are reemerging as cutting edge devices. This revival is due to the development of new classes of efficient materials that use low driving temperatures, which were previously not achievable, and maintain high cooling output while employing only water as the working fluid^7–9^.

Adsorption driven chillers (ADCs) function through the endothermic process of evaporation (Supplementary Fig. 1)^10,11^. In these sealed devices, water, as the most common fluid, is stored at a heat exchanger in the liquid phase. The first (working) step consists of evaporation, which induces the cooling effect (desired cooling temperature). This is induced by the presence of a porous active material which adsorbs water vapor from a water reservoir. As adsorption is an exothermic process, this step generates heat which must be dissipated (heat rejection temperature). The adsorbent must subsequently be regenerated after it has been saturated. This is the crucial step regarding efficiency and it is performed by heating the active material to desorb water (driving temperature). It is the only step that consumes thermal energy in the cycle. The desorbed water is condensed at the heat exchanger again, where heat is generated and has to be dissipated (back cooling temperature). Heat rejection and back cooling is typically performed at the same temperature level as they are both cooled against the outside temperature. Using two or more of these devices working phase-shifted enables continuous cooling. The efficiency of this cycle depends highly on the amount of water that can be exchanged between the adsorption and the desorption stage. In order to achieve high-power density, short cycling times are mandatory which require extended cycling stability of the material.

Conventional adsorbents require either very high desorption temperatures (e.g., hydrophilic zeolites like NaA or 13X) or feature an unwanted linear isotherm shape (e.g., silica gel) that limits the exchangeable amount of water^12^. In contrast, an S-shaped water adsorption isotherm can be seen as beneficial due to the fact, that the system is switched between the working and regeneration cycle by adjusting the temperature, which leads to a change of the relative pressure (*p*_water_/*p*_saturation_ or *p*/*p*_0_). With smaller temperature differences between the adsorption and desorption step, the switching process becomes faster and more energy efficient since less energy is consumed to overcome the thermal capacities (adsorbent, binder, heat exchanger, piping…) while heating and cooling.

The choice for the optimal material in such applications depends on a number of variables, such as the desired cooling temperature, the possibility to reject the heat of adsorption, and condensation. In addition, to improve the efficiency of the adsorbent an increase of its uptake capacity and a decrease of the driving temperature are both targeted. A lower driving temperature has two major benefits. First, it allows the utilization of new heat sources (e.g. district heating, geothermal heating, data centers) and second, the available energy is more efficiently exploited (Supplementary Note 1).

ADC systems can be categorized according to the needed driving temperature as high (>200 °C), medium (120–200 °C), low (80–120 °C) and ultra-low (<80 °C) temperature systems. Low and ultra-low temperature systems require the lowest driving energy, thus extending the range of usable energy source, and they can be realized, for example, by using metal-exchanged aluminum phosphates such as SAPO-34 (~90 °C)^13,14^ or metal-organic frameworks (MOFs)^15–34^. The water sorption properties of the latter family of adsorbent are attractive for this application, however as one of the many prerequisites for a long term use under real conditions high stability is mandatory but only a few suitable MOFs have been reported. Especially some aluminum containing compounds such as MIL-160 (90 °C)^24,25^, CAU-10-BDC (70 °C)^26,27^ and Al-fum (90 °C)^28,33,34^ have been tested for their applicability in ADC systems. A driving temperature of 65–70 °C has been the lower limit for such materials until now, which was realized by CAU-10-BDC and MIP-200^16,26^. The driving temperature of a MOF can be tuned by variation of the linker molecule, which has been demonstrated using a series of CAU-10 type structures ([Al(O_2_C-R-CO_2_)(OH)]; R = aryl or heterocycle). The replacement of isophthalate by the more hydrophilic furandicarboxylate ions led to the CAU-10 type compound known as MIL-160, which exhibits a steep water uptake at a lower relative humidity value (*p*/*p*_0_ = 0.08, 25 °C) and consequently requires a higher driving temperature. Damasceno Borges et al. predicted that the thiophene dicarboxylate (TDC^2−^) analog of CAU-10 should be more hydrophobic and thus it is expected to be applicable at lower driving temperatures compared with CAU-10-BDC^35^. Even if these differences are rather small (+0.02 *p*/*p*_0_), every step towards reducing the driving temperature, which is accomplished by shifting the uptake to higher *p*/*p*_0_ values, can potentially lead to an enormous amount of additional heat sources that can be used in ADC applications^36^. Following this concept we discovered a highly stable Al-MOF using H_2_TDC as the linker. The material was successfully obtained under green, potentially scalable synthesis conditions with high yield and water as the sole solvent. Our results emphasize its outstanding ADC properties at very low driving temperatures.

## Results

### Green synthesis and structure determination

The green, high-yield synthesis of the compound, [Al(C_6_H_2_O_4_S)(OH)] ∙ *x*H_2_O named CAU-23, was achieved by a reaction between AlCl_3_ and NaAlO_2_ as the metal sources, and sodium thiophenedicarboxylate (Na_2_TDC) as the linker source. Upon mixing, a white X-ray amorphous precipitate is formed which subsequently crystallizes under reflux conditions at ambient pressure. Thus 4.30 g H_2_TDC (25 mmol) was mixed with 2.0 g (50 mmol) sodium hydroxide in 100 mL distilled water until a clear solution of Na_2_TDC was obtained. After adding 18.75 mL of aqueous aluminum chloride solution (1 mol/L, 18.75 mmol), and 12.5 mL of aqueous sodium aluminate solution (0.5 mol/L, 6.25 mmol), the slurry was stirred under reflux conditions for 6 h, and then filtered off and dried at 100 °C for 4 h. Only an aqueous sodium chloride solution is formed as the other reaction product. After an additional washing step with 200 mL water under stirring and reflux, filtration and drying, 4.5 g of a white powder was obtained (84% yield based on H_2_TDC). Thermogravimetric analysis, infrared spectroscopy, elemental analysis, and nitrogen sorption measurements are in good agreement with the expected values for the ideal composition (Supplementary Figs. 2–4, Supplementary Table 2). Temperature-dependent powder X-ray diffraction measurements and thermogravimetric analysis demonstrate thermal stability of CAU-23 in air up to 400 °C (Supplementary Fig. 5).

**Fig. 1:**
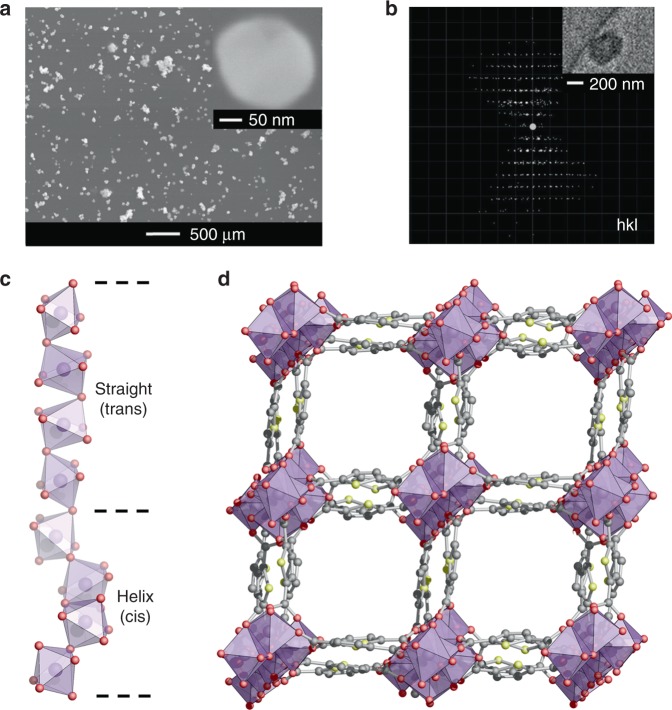
Determination and details of the crystal structure of CAU-23. **a** SEM image of CAU-23. **b** Reconstructed 3D reciprocal lattice from cRED data of a nano-sized single-crystal (inset) of CAU-23. **c** The repetition of cis and trans corner-sharing AlO_6_ polyhedra forming the inorganic building unit of CAU-23. **d** The full structure of CAU-23 projected along [010]; water molecules are omitted for clarity

Acquisition of single crystal X-ray diffraction data of adequate quality for structure determination was not possible due to the nano-scale particle size of only 200 nm determined by scanning electron microscopy (SEM, Fig. 1a). The large unit cell, peak overlapping and the complicated structure also prevented structure determination from powder X-ray diffraction data.

On the other hand, 3D single crystal electron diffraction can be obtained from powder samples and used for determination of unknown structures^37^. Therefore, we performed continuous rotation electron diffraction (cRED, Fig. 1b) for structure determination of CAU-23. The data collection was challenging because of the small crystal sizes (200 × 200 × 100 nm^3^) and weakly scattering elements in CAU-23. To push the size limit of MOF crystals and reduce electron beam damage, we applied low-dose illumination and fast data acquisition to collect cRED data using a Timepix hybrid electron detector with high sensitivity, low background, and fast readout time^38^. Each high resolution (1.13 Å) cRED dataset was collected in ca 3 min, from which the unit cell parameters and space group were determined. The structure was solved and refined from the cRED data with the resolution of 1.13 Å, and all framework atoms were located with high precision and reasonable agreement values (Supplementary Fig. 6b, Supplementary Table 3). It is to the best of our knowledge among the smallest ever used MOF crystals applying TEM electron diffraction for structure solution and refinement. Although the cRED data were clearly sufficient for determining the framework structure, it was not possible to locate water molecules in the pores. Therefore, structure refinement was successfully performed against PXRD data for a dry and a wet sample using the model from cRED as the starting model (Supplementary Figs. 7–8, Supplementary Table 4).

Both forms of CAU-23 crystallize in a non-centrosymmetric space group forming a chiral structure (Fig. 1c, d). The rod-shaped inorganic building unit is formed by alternating units of four consecutive *trans* and *cis* corner-sharing AlO_6_ polyhedra, each, which correspond to straight and helical sections, respectively, in the Al–O chain. Interestingly, these sections resemble a combination of the inorganic building units observed in MIL-53 (only *trans*) and CAU-10 (only *cis*) (Supplementary Fig. 10). Square channels are formed through the interconnection by TDC^2−^ ions and propagate along the *b*-axis, with a side length of 7.6 Å. A detailed topological description is provided in the Supplementary Note 2. The MOF exhibits permanent porosity and N_2_ sorption measurements resulted in a BET area of 1250 m^2^/g, which corresponds well to the theoretical accessible surface area of 1330 m^2^/g.

### Water sorption behavior

**Fig. 2:**
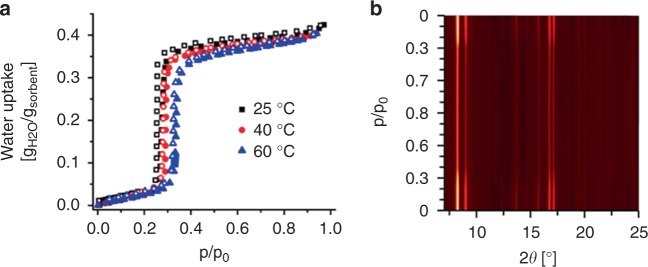
Water sorption properties of CAU-23. **a** Water sorption isotherms recorded at three different temperatures (filled symbols = adsorption; empty symbols = desorption). **b** PXRD patterns of CAU-23 at different relative water pressure values

Preliminary water stability tests at room and elevated temperatures did not show any degradation of the crystallinity. Hence detailed water adsorption studies were carried out. The water sorption isotherms of CAU-23 were recorded at three different temperatures (Fig. 2a). The measurements show an S-shaped isotherm without hysteresis with a steep uptake of 0.375 *g*_H2O_/*g*_sorbent_ up to *p*/*p*_0_ = 0.33 at 25 °C. With increasing temperature the sharp uptake shifts to higher relative pressure and the uptake capacity is lowered, which is expected for microporous compounds (0.351 *g*_H2O_/*g*_sorbent_ at *p*/*p*_0_ = 0.29 and 40 °C; 331 *g*_H2O_/*g*_sorbent_ and *p*/*p*_0_ = 0.33 at 60 °C).

PXRD measurements carried out on CAU-23 to investigate the influence of relative humidity at 25 °C show only minor shifts in peak positions and only changes in the relative intensities due to pore filling and evacuation (Fig. 2b).

A detailed discussion on the crystal structure–water sorption property relationship in Al-MOFs is presented in Supplementary Note 4. Overall, both the linker, i.e., size, geometry, and hydrophilicity, and the exact structure of rod-like IBUs determine the properties of the Al-MOF. A future design of better MOFs for ADC applications seems hard to accomplish and hence exploratory investigations such as the one presented here will still be necessary.

To establish a deeper understanding of the water uptake mechanism the water adsorption isotherm of CAU-23 was simulated using force-field-based grand Canonical Monte Carlo (GCMC) simulations at 25 °C.

**Fig. 3:**
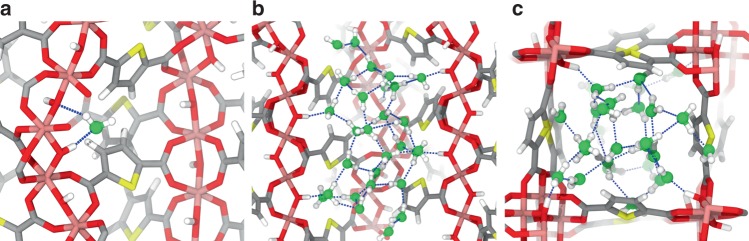
Preferential arrangement of the adsorbed water molecules within the channel of CAU-23. **a** The very first adsorbed water molecule interacting with two adjacent µ-OH sites forming strong hydrogen bonds. **b** Aggregation of hydrogen-bonded water molecules within the channel of CAU-23 plotted along the cross-section of the channel. **c** Top view of the water loaded channel

The experimental adsorption isotherm is relatively well reproduced, particularly the steep increase in the water uptake at *p*/*p*_0_ in the range of 0.20–0.30 (Supplementary Fig. 13). Furthermore, we explored the adsorption mechanism at the microscopic level. The adsorption of water occurs initially between two hydroxyl groups (μ-OH) of the inorganic building unit (Fig. 3a). On analyzing the radial distribution functions (RDFs) of the corresponding intermolecular pairs (Supplementary Fig. 14), we also found that the adsorbed water molecules (O_w_) interact more strongly with µ-OH sites than with other adsorbed water molecules (O_w_) as substantiated by (i) a shorter mean O_μ-OH_···O_w_ distance of 2.72 Å vs. O_w_···O_w_ distance of 2.83 Å, and (ii) a shorter mean H_μ-OH_···O_w_ distance of 1.75 Å vs. O_w_···H_w_ distance of 1.88 Å. In addition, the relatively higher intensity of the H_μ-OH_···O_w_ over the O_μ-OH_···H_w_ RDF plots (Supplementary Fig. 14) further suggests that μ-OH groups favorably act as H-donors while forming hydrogen bonds. A detailed statistical breakdown on the occurrences of different hydrogen bond configurations (Supplementary Figs. 15–16) supports that the H_μ-OH_···O_w_ configuration is the driving force that initiates the adsorption process in CAU-23. The population of adsorbed water molecules continues to rise until corresponding to a monotonic increment of the water molecules in the region nearby the µ-OH sites (Supplementary Fig. 17). Upon covering these primary hydrophilic sites, there is a sudden increase in the water content above *p*/*p*_0_ ≈ 0.30 and the water molecules tend to occupy the 1D square-shaped channels. The entire pore system of CAU-23 is almost completely filled at a relative pressure of *p*/*p*_0_ ≈ 0.35. This pore filling mechanism is characterized by the aggregation of water molecules interacting with each other through strong hydrogen bonds as evidenced in Fig. 3b, c. To this end, the fraction of the hydrogen bonds formed among the adsorbed water molecules to the total number of hydrogen bonds drastically increases from ~20% to ~90% as the relative pressure (*p*/*p*_0_) increased from 0.20 to 0.35, respectively (Supplementary Fig. 15). The average number of hydrogen bond connections associated with a single water molecule is ~3.65—a number very close to the one of bulk water and it is also consistent with our previous findings in other MOFs and related materials^19,22,39–41^.

### Stability tests of CAU-23 at working conditions

**Fig. 4:**
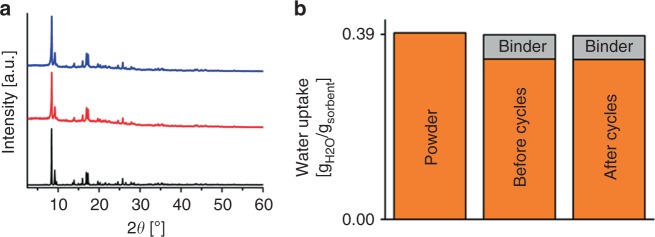
Proof of integrity of coated CAU-23 before and after 5000 cycle stability measurement. **a** PXRD patterns of CAU-23 before (red) and after 5000 sorption cycles (blue). A calculated pattern (black) based on the crystal structure is also provided. **b** Gravimetric determination of the water uptake capacity of CAU-23 coatings before and after the 5000 cycle stability measurement in comparison with the value of the pure nanoscale powder

To confirm the promising properties of CAU-23 in ADC applications, long-term cycling tests were carried out in order to prove the required high stability at working conditions. Hence, CAU-23 was subjected to an extended test of 5000 water sorption cycles^42^. An aluminum sheet was coated with the nanocrystalline powder and the sample was cycled between 20 and 120 °C in a saturated water atmosphere to promote fast adsorption and desorption (see method section for details). Cycling stability was confirmed by PXRD and water adsorption capacity measurements through a comparison of the original coated sample and the sample after the tests (Fig. 4a, b, Supplementary Fig. 19, Supplementary Tables 8–9). Le Bail fits of the PXRD data demonstrate persistent crystallinity of the compound. The water uptake capacities are very consistent and within the error limit of the measurement. Uptakes of 0.393, 0.389, and 0.387 *g*_H2O_/*g*_sorbent_ were recorded for the pure nanoscale powder and the coating before and after testing, respectively, while taking the amount of binder into account. The volumetric uptake capacity of a MOF/binder (83.3/16.7 wt%) composite was determined to be 0.14 *g*_H2O_/cm³composite (composite density 0.46 g/cm³, Supplementary Table 10). It should be kept in mind that although MOFs are intensively discussed for water adsorption applications, such long-term stability studies involving a few thousand cycles have rarely been reported^27,33^. Thus the water adsorption properties of CAU-23 in combination with its high stability prompted us to determine its performance at low and ultra-low driving temperatures.

### ADC calculations for CAU-23

**Fig. 5:**
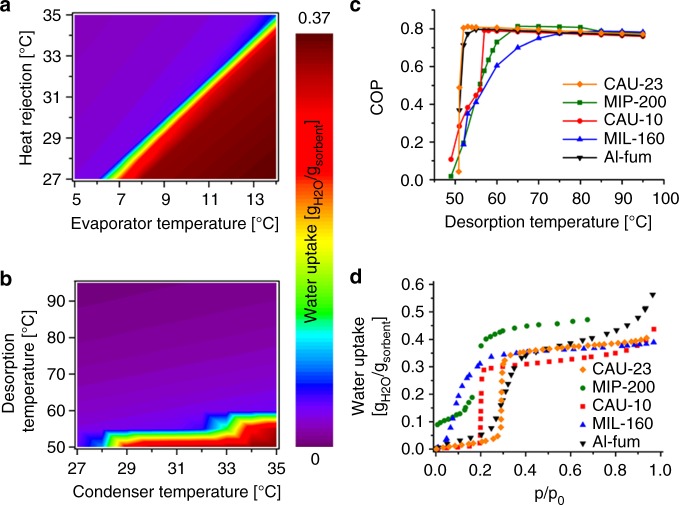
Calculation of adsorption driven chiller temperature boundaries for CAU-23 and coefficient of performance values for cooling in comparison with selected state of the art materials. Calculated loading of CAU-23 for different temperatures used in an ADC setup for adsorption **a**, and desorption **b**, cycles. **c** Calculation of the COP values for different driving temperatures (assumed desired cooling of 10 °C and back cooling temperature of 30 °C). **d** Water adsorption curves at 40 °C of selected compounds

The various temperatures within the ADC unit, i.e., the desired cooling, back cooling/heat rejection, and driving temperatures, define the working conditions of the ADC setup. The efficiency of ADCs strongly depends on these temperatures, and so an understanding of the properties of the compound with respect to the set of temperatures is required. The heat of adsorption of the water molecules was first determined from the adsorption isotherms collected at several temperatures (Supplementary Fig. 20). The resulting moderate value of −48.2 kJ/mol strongly suggests that the water desorption should be easily achieved at relatively low driving temperatures since it is only slightly higher than the evaporation enthalpy of water 44.19 kJ/mol (Supplementary Fig. 21)^43^. In addition, the loading of the compound during adsorption (Fig. 5a) and desorption cycles (Fig. 5b) was further calculated from the sorption isotherms. To identify suitable temperature boundaries (*T*_driving_/*T*_condenser/heat rejection_/*T*_evaporator_) where the use of the full sorption capacity of CAU-23 of >0.35 *g*_H2O_/*g*_sorbent_ is possible, the temperatures that lead to a complete adsorption in the adsorption step (Fig. 5a, dark red area) and a complete desorption in the desorption step (Fig. 5b, dark blue area) must be chosen. A selection of possible temperature boundaries with a driving temperature of 60 and 55 °C is given in the Supplementary Fig. 22. These results demonstrate that at low desired cooling temperatures above 7 °C the full loading capacity (>0.35 *g*_H2O_/*g*_sorbent_) can be used but with the drawback of a low heat rejection temperature below 30 °C. With higher heat rejection temperatures above 30 °C, cooling temperatures of 10 °C can still be obtained, which is low enough, for example, to run a domestic cooling system. The greatest advantage of this particular compound is its desorption behavior. At 60 °C, or even lower if back cooling temperatures are below 30 °C, the material can be emptied completely and reset to the initial state. So with a temperature set of e.g., 60/30/10 °C (driving temperatures /back cooling or heat rejection/ desired cooling) the full loading capacity can be used.

To compare the efficiency, a material related coefficient of performance for cooling (COP) was calculated (see Methods) according to the methodology by de Lange et al. and Ernst et al.^8,44^ for CAU-23 and selected materials (MIP-200, CAU-10-H, the Al-fum and MIL-160)^16,24,26,28^ that have been proposed in the past as outperforming conventional adsorbents (Fig. 5c, Supplementary Fig. 23). The Zr-MOF MOF-801, also known as Zr-fum^45^, is not discussed in this study since its long term cycling stability has not been demonstrated yet^22,23,31^.

Even at the temperature boundaries of 30 °C for back cooling and 10 °C for desired cold, CAU-23 outperforms other best-in-class adsorbents not only in terms of its very high COP of 0.8 but, more importantly, by maintaining this high COP even at very low-desorption temperatures of nearly 50 °C. Only Al-fum shows similar theoretical COP values but its water sorption isotherm deviates distinctly from the optimal S-shape form and exhibits a significant hysteresis, which will lower the efficiency in devices (Fig. 5d). This makes CAU-23, to the best of our knowledge, the material with the lowest reported driving temperature (<60 °C) combining high capacity (0.37 *g*_H2O_/*g*_sorbent_) and proven long-term stability (>5000 cycles). The low driving temperature allows the utilization of heat sources that are currently not used and simultaneously the available energy is more efficiently used leading to an outstanding efficiency. The next step, the design, construction, and testing of a suitable prototype giving meaningful conclusions at the technology level is by far more complex. So far the proof of concept of this technology was validated in the laboratory by some of the authors^26,28^. Based on the previous studies, a good correlation between lab-based calculations of performance and the experimental performance of the coated heat exchanger (the heart of an ADC) was found (for details see SI).

CAU-23 has been demonstrated to be an ideal material for ultra-low temperature adsorption driven chillers. It allows to lower the required driving temperatures of such devices to 60 °C, while possessing high uptake capacities of 0.37 *g*_H2O_/*g*_sorbent_ and providing a low cooling temperature of 10 °C. The most significant advantage of CAU-23 is that the driving temperatures of 60–70 °C can be easily achieved without any loss of performance, paving the way for more efficient use of waste heat or solar heat. CAU-23 outperforms all other microporous materials considered so far for ADC applications, in terms of having a low driving temperature while exhibiting high-water uptake capacity, outstanding coefficient of performance values at low driving temperature and proven excellent stability. The green synthesis, which can potentially be scaled up easily, opens viable perspectives towards applications at the industrial-level.

## Methods

### Standard measurements

Elemental analysis was carried out on an Elementar vario Micro cube (CHNS). Thermogravimetric measurements were recorded in air on a Linseis STA PT 1600 (heating rate = 4 K min^−1^, gas flow = 20 mL/min). Infrared spectra were recorded on a Bruker Alpha-P IR spectrometer (drying of the sample was carried out at 100 °C for 16 h). Scanning electron microscopy micrographs were collected using a JEOL JSM-7000F, equipped with an Everhart-Thornley detector. Temperature dependent powder X-ray diffraction (T-PXRD) patterns were recorded on a Stoe Stadi P Combi diffractometer in transmission geometry equipped with Mo-K_α1_ radiation, a curved germanium monochromator and a linear MYTHEN detector with an aperture angle of 17° and a furnace. Nitrogen sorption experiments were carried out on a BEL Japan Inc. Belsorpmax at 77 K. The sample was activated at 150 °C for 16 h under reduced pressure (<0.1 mbar).

### Single crystal electron diffraction using cRED

The sample of CAU-23 was crushed in a mortar and dispersed in ethanol. A few drops of hydrochloric acid (0.1 mol/L) were added to break the agglomeration of the CAU-23 nanocrystals. The suspension was then treated by ultrasonication for about 30 s. Three droplets from the suspension were applied on a lacey carbon TEM grid (Okenshoji Co., Ltd, Tokyo, Japan). cRED data were collected on a 200 kV JEOL JEM-2100 LaB_6_ TEM at room temperature. The rotation speed of the goniometer, exposure time, spot size, and camera length were 0.45°/s, 1 s, 2, 40 cm, respectively. Video frames of electron diffraction patterns were recorded by a high-speed hybrid electron camera (Timepix QTPX-262). The total rotation angle of each data set ranged between 70 to 90° and the total collection time was between 2.5 and 3.5 min.

### cRED data processing and structure determination

cRED data were first processed by using REDp^46,47^ for initial determination of unit cell and space group. For structure solution, cRED data were then processed by Dials^48^, where the instrumental parameters (rotation axis, beam position, and beam direction), unit cell, orientation matrix, and intensity profiles were refined and the intensities were integrated. Four data sets were merged together by XSCALE in XDS^49,50^ and used for structure solution and refinement by SHELX. Isotropic structure refinement was performed against the cRED data without adding any restraints. The crystallographic data of CAU-23 are given in Supplementary Table 2.

### High resolution powder X-ray diffraction measurements

The high resolution powder X-ray diffraction data were  recorded at beamline I11 at Diamond Light Source Didcot, UK^51^. A 0.5 mm capillary was filled, evacuated and heated to 100 °C for 2 h and then sealed. The measurements were performed on a setup consisting of 9 Dectris^®^ Mythen 2 detectors with an overall opening angle of 90° 2θ at room temperature.

### Rietveld refinement

Rietveld refinement was subsequently performed by TOPAS 6 against synchrotron PXRD data^52^. All atoms were refined with isotropic displacement parameters. The ligand was modeled using a Z-matrix to constrain some of the geometry. Aluminum–oxygen distances and oxygen–aluminum–oxygen angles were allowed to be refined with bond distance and angle restraints. The positions of water molecules in the pores were established by simulated annealing using antibump restraints, followed by refinement with restraints. Hydrogen atoms were excluded in the Rietveld refinement.

### Water sorption experiments

The water adsorption was studied by measuring isotherms at 25, 40, and 60 °C with a Quantachrome VStar. Prior to the measurements the MOF was outgassed at 120 °C under vacuum in a MasterPrep^®^ Degasser.

### Computational methods

The CAU-23 crystal structure was further geometry optimized—keeping the lattice parameters constant— by applying a tighter convergence criteria for atomic displacements (<2 ∙ 10^−5^ bohr), maximum forces acting on individual atoms (2 ∙ 10^−5^ hartree/bohr), and also for SCF energies (10^−8^). These DFT calculations were performed using the general gradient approximation (GGA) to the exchange-correlation functional according to Perdew–Burke–Ernzerhof (PBE)^53^ in a combination of Grimme’s DFT¬D3 semi-empirical dispersion corrections^54,55^. Triple-ζ plus valence polarized Gaussian-type basis sets (TZVP-MOLOPT) were considered for all atoms, except for the Al centers, where short ranged double-ζ plus valence polarization functions (DZVP-MOLOPT) were employed^56^. The interaction between core electrons and valence shells of the atoms were described by the pseudopotentials derived by Goedecker, Teter, and Hutter (GTH)^57-59^. The auxiliary plane wave basis sets were truncated at 400 Ry. Single point energy calculations were performed to extract the atomic partial charges of CAU-23 applying the Restrained Electrostatic Potential (RESP) fitting strategy for the periodic system as implemented in the CP2K code (Supplementary Fig. 16 for atom types).

The DFT geometry optimized model of CAU-23 was further employed in Grand Canonical Monte Carlo (GCMC) calculations to determine the water adsorption properties of the MOF at 25 °C using the Complex Adsorption and Diffusion Simulation Suite (CADSS) code^60^. We considered a simulation box of 18 conventional unit cells (3 × 2 × 3) of CAU-23 maintaining atoms at their initial positions.

The water molecules were described by the TIP4P/2005 potential model corresponding to a microscopic representation of four Lennard–Jones (LJ) sites (Supplementary Table 4)^61^. This potential model was demonstrated to lead to a good agreement between the experimental and simulated adsorption isotherms for a series of MOFs^15,16,26,28^, especially at very low pressure, which is where the adsorption domain is most affected by the choice of the potential model. The interactions between the guest water molecules and the MOF structure were described by a combination of site-to-site LJ contributions and Coulombic terms. A mixed set of the universal force field (UFF)^62^ and DREIDING force field^63^ parameters were adopted to describe the LJ parameters for the atoms in the inorganic and organic part of the framework, respectively (Supplementary Table 5). However, following the treatment adopted in other well-known force fields^64,65^, the hydrogen atoms of the µ-OH moieties and organic linkers, as well as Al and S atoms are allowed to interact with the adsorbate water molecules via the coulombic potential only as justified in previous studies on similar Al-based MOF topologies^25,35^. Short-range dispersion forces were truncated at a cutoff radius of 12 Å while the interactions between unlike force field centers were treated by means of the Lorentz–Berthelot combination rule. The long-range electrostatic interactions were handled using the Ewald summation technique. For each point in the adsorption isotherm, a typical 2 ∙ 10^8^ MC steps for equilibration and 3 ∙ 10^8^ MC steps have been used for production runs. The adsorption enthalpy at low coverage (∆H) was calculated through the configurational-bias Monte Carlo simulations performed in the NVT ensemble using the revised Widom’s test particle insertion method^66^. In addition, in order to gain insight into the configurational distributions of the adsorbed species in CAU-23, some additional data were calculated at different pressure including the hydrogen bond networks and the radial distribution functions (RDF) of the intermolecular atomic pairs of the guests and the host. The detection of hydrogen bonds has been conducted by assuming the following criteria: (i) distance between donor and acceptor oxygen centers (D−A) is shorter than 3.5 Å, and (ii) the corresponding D-H-A angle—formed by the intramolecular O−H vector of the donor molecule, and the intermolecular H−O vector of donor and acceptor molecules—is greater than 120°.

### Cycling stability test

Advanced long-term hydrothermal stability to 5000 full cycles was tested within a custom-made cycle apparatus as described elsewhere^42^. The sample was alternatingly tempered at 120 °C and 20 °C, respectively, under a pure water vapor atmosphere. Prior to and after the cycle experiment, the sample was subjected to a PXRD measurement to identify possible degradation of the framework. For the 5000 cycle experiment, the MOF was coated on a 50 × 50 mm aluminum sheet using a coating recipe described elsewhere (binder mass = 15 wt%)^67^.

Powder X-ray diffraction (PXRD) analysis was performed on a Bruker D8 Advance with DaVinci™ design, using Cu-K_α_ radiation from a Cu anode tube at 40 kV/40 mA with a Ni filter in Bragg–Brentano geometry. An MRI TC-humidity chamber, coupled to a humidified nitrogen flow generated by an Ansyco^®^ humidifier, was used for controlled humidity PXRD experiments.

### Calculations of water sorption properties for ADC applications

To assess the potential of CAU-23 in adsorption heat transformation, the measured water adsorption data were fitted using a weighted-dual site Langmuir approach (wDSL)^67,68^.

1 $$X\left( {{\mathrm{p}},{\mathrm{T}}} \right) = X_{\mathrm{L}}\left( {1 - w\left( {{\mathrm{p}},{\mathrm{T}}} \right)} \right) + X_{\mathrm{U}}\left( {{\mathrm{p}},{\mathrm{T}}} \right)w\left( {{\mathrm{p}},{\mathrm{T}}} \right)$$

2 $$X_L\left( {{\mathrm{p}},{\mathrm{T}}} \right) = X_{L,\infty }\frac{{b_L{\mathrm{p}}}}{{1 + b_L{\mathrm{p}}}}$$

3 $$X_U\left( {{\mathrm{p}},{\mathrm{T}}} \right) = X_{U,\infty }\frac{{b_U{\mathrm{p}}}}{{1 + b_U{\mathrm{p}}}} + b_H{\mathrm{p}}$$

4 $$b_\alpha = b_{\alpha ,\infty }\exp \left( {\frac{{E_\alpha }}{{R{\mathrm{T}}}}} \right),\alpha = {\mathrm{L}},{\mathrm{U}},{\mathrm{H}}$$

5 $$w\left( {{\mathrm{p}},{\mathrm{T}}} \right) = \left( {\frac{{\exp \left( {\frac{{\ln \left( {\mathrm{p}} \right) - \ln \left( {p_{{\mathrm{step}}}\left( {\mathrm{T}} \right)} \right)}}{{\sigma \left( {\mathrm{T}} \right)}}} \right)}}{{1 + \exp \left( {\frac{{\ln \left( {\mathrm{p}} \right) - \ln \left( {p_{{\mathrm{step}}}\left( {\mathrm{T}} \right)} \right)}}{{\sigma \left( {\mathrm{T}} \right)}}} \right)}}} \right)^\gamma$$

6 $$\sigma ({\mathrm{T}}) = \chi _1\exp \left( {\chi _2\left( {\frac{1}{{T_0}} - \frac{1}{{\mathrm{T}}}} \right)} \right)$$

7 $$p_{{\mathrm{step}}}\left( {\mathrm{T}} \right) = p_{{\mathrm{step}},0}\exp \left( {\frac{{ - H_{{\mathrm{Step}}}}}{R}\left( {\frac{1}{{T_0}} - \frac{1}{{\mathrm{T}}}} \right)} \right)$$

The water uptake at a certain pressure and temperature *X(p, T)* is calculated from two Langmuir-terms (*X*_*L*_ and *X*_*U*_), representing the adsorption before and after the step in the uptake. *w(p, T)* is a weighting function that depends on the pressure *p*, the temperature *T* and the pressure *p*_step_ at which the uptake step occurs. Further symbols *X*_*∞*_, *b*_*α*_, *E*_*α*_, and *X*_*1,2*_ represent fit parameters^68,69^.

This model was then used for the calculation of uptake capacity as a function of the temperatures applied for adsorption, desorption, condensation and evaporation.

The heat of adsorption was calculated from measured adsorption isotherms, as well as from the thermodynamic fit using the van’t Hoff Eq.^70^:

8 $$\frac{{{\mathrm{\Delta }}H_{ads}\left( {X,T} \right)}}{{RT^2}} = - \left( {\frac{{\partial \ln p}}{{\partial T}}} \right)_{X\left( {p,T} \right)}$$

### COP calculation

The coefficient of performance (COP) for cooling can be defined as the ratio of evaporation enthalpy of the liquid phase and consumed heat for the desorption process:^8,44^

9 $${\mathrm{COP}}_C = \frac{{Q_{{\mathrm{evap}}}}}{{Q_{{\mathrm{des}}} + Q_{{\mathrm{IH}}}}}$$

Herein in the numerator the evaporation enthalpy of water is used (44.19 kJ/mol)^44^. In the denominator the amounts of heat to apply for desorption (*Q*_des_) and isosteric heating (*Q*_IH_) of the adsorbent are summed up.

The amounts of heat can be calculated from energy balances:

10 $$dQ_{{\mathrm{IH}}} = m_{{\mathrm{ads}}} \cdot \left( {c_{{\mathrm{p,ads}}} + X_{{\mathrm{max}}}c_{{\mathrm{p,fl}}}} \right){\mathrm{dT}}$$

11 $$dQ_{{\mathrm{des}}} = m_{{\mathrm{ads}}} \cdot \left( {c_{{\mathrm{p,ads}}} + X\left( {p,T} \right)c_{{\mathrm{p,fl}}}} \right)dT - m_{{\mathrm{ads}}}q_{{\mathrm{st}}}(T)dX$$

12 $$Q_{{\mathrm{evap}}} = m_{{\mathrm{ads}}} \cdot \left( {{\mathrm{\Delta }}h_{{\mathrm{vap}}}\left( {T_{evap}} \right) - c_{{\mathrm{p,g}}}\left( {\bar T - T_{{\mathrm{evap}}}} \right)} \right)(X_{{\mathrm{max}}} - X_{{\mathrm{min}}})$$

Herein *m*_*ads*_ refers to the adsorbent mass, *c*_*p,ads*_, *c*_*p,fl*_, and *c*_*p,g*_ to the isobaric heat capacities of adsorbent, water and water vapor, $\bar T$ to the arithmetic mean temperature during desorption.

13 $$\bar T = 0.5\left( {T_{{\mathrm{des,max}}} + T_{{\mathrm{des,min}}}} \right)$$

The uptake *X(p, T)* is calculated from either the wDSL approach described within in the paper or using the approach of Dubinin–Astakhov, depending on which fits best.

Within the latter, briefly described, an adsorption potential *A(p, T)* is defined:

14 $$A\left( {p,T} \right) = - RT{\mathrm{ln}}\left( {\frac{p}{{p_s\left( T \right)}}} \right)$$

Further, the water uptake is expressed as an adsorbed volume *W* of liquid water by dividing the mass specific uptake by the liquid density of the adsorptive:

15 $$W = \frac{{X\left( {p,T} \right)}}{{\rho _L\left( T \right)}}$$

The plot *W(A)* is referred to as characteristic curve and can be described for the adsorption of water on many conventional adsorbents by a semi-empirical approach:

16 $$W\left( A \right) = W_0 \cdot \exp \left( { - \left( {\frac{A}{{E_A}}} \right)^n} \right)$$

Herein, *W*_*0*_*, E*_*A*_, and *n* are parameters free to fit to experimental data. As proposed by for instance de Lange et al. *W(A)* can be defined in sections^8^.

Within the Dubinin–Astakhov approach, the isosteric heat of adsorption $q_{{\mathrm{st}}}$ can be calculated as follows:

17 $$q_{{\mathrm{st}}} = {\mathrm{\Delta }}h_{{\mathrm{vap}}}\left( T \right) + A\left( T \right)$$

Using the above described set of equations, the COP for cooling was calculated for a back cooling/ heat rejection temperature of 30 °C, and desired cold temperature of 10 °C and a variation of driving temperatures lower than 95 °C. As suggested by de Lange et al. and for the sake of comparability, the capacity of the adsorbent *c*_*p,ads*_ was assumed to 1 kJ/kg^8^.

## Supplementary information

Supplementary Information

Transparent Peer Review File

## Data Availability

The X-ray crystallographic data for CAU-23 have been deposited at the Cambridge Crystallographic Data Centre (CCDC, free for charge at https://www.ccdc.cam.ac.uk) under deposition number CCDC 1878820. Further data that supports the findings of this study are available from the corresponding authors upon reasonable request.
