# Supplementary material for: A metal–organic framework for efficient water-based ultra-low-temperature-driven cooling
Source: Nat Commun. 2019 Jul 9;10:3025. doi: 10.1038/s41467-019-10960-0 (PMC6616384; doi:10.1038/s41467-019-10960-0)
Supplement: Supplementary file 1 — Supplementary Information [file 41467_2019_10960_MOESM1_ESM.pdf]

Supplementary Information

A metal-organic framework for efficient water-based ultra-low-temperature-driven cooling

Lenzen et al.

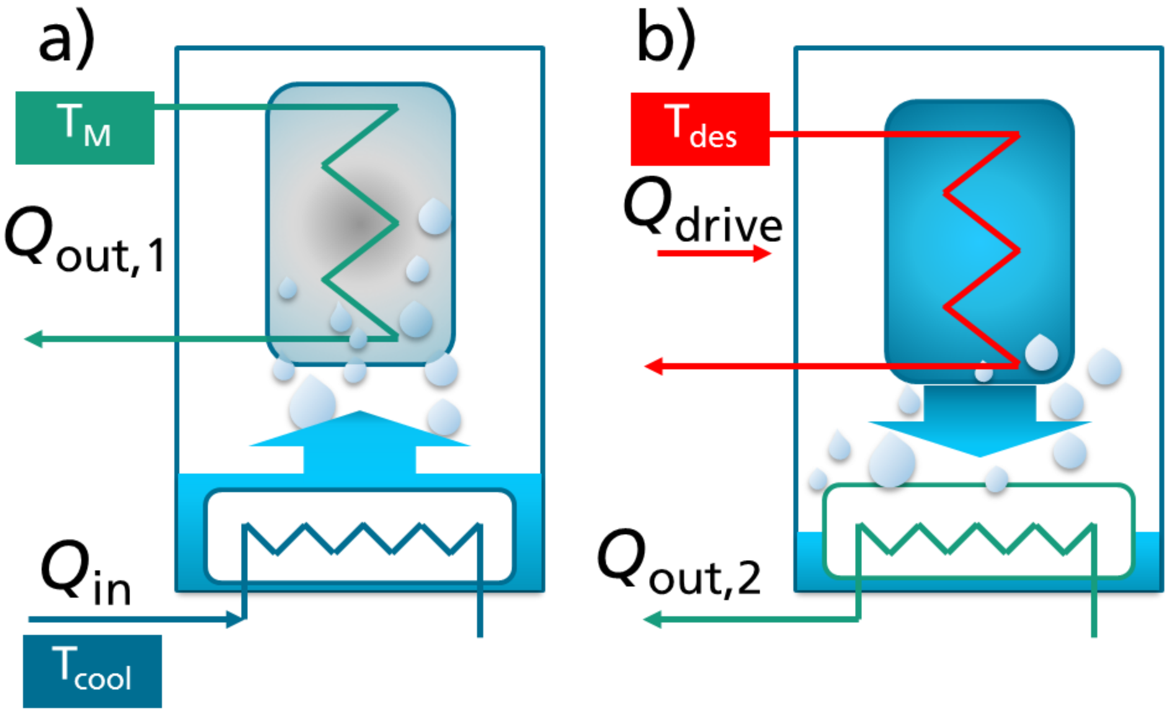

Supplementary Figure 1: Scheme of the working (a) and regeneration (b) cycle of an adsorption driven chiller device with T_{cool} as desired cooling temperature (blue) T_M as heat rejection and back cooling temperature (green) and T_{des} as driving temperature (red).

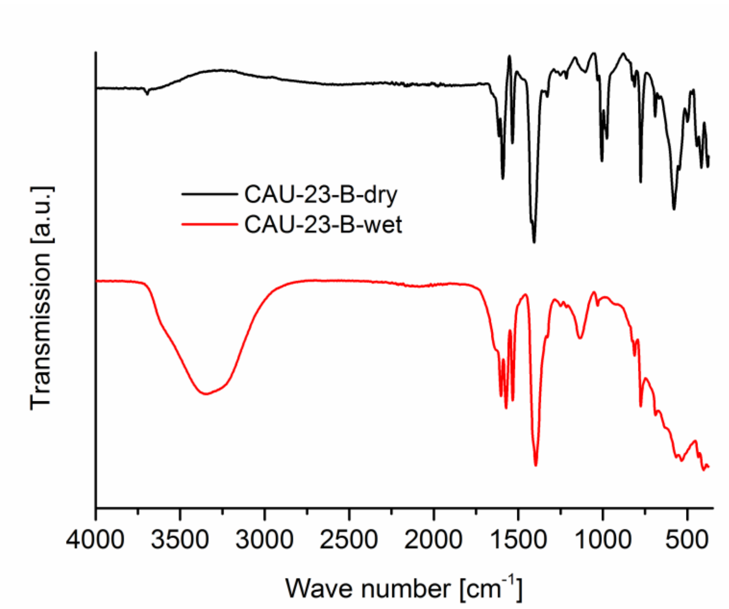

Supplementary Figure 2: IR spectra of CAU-23 in dry and wet state.

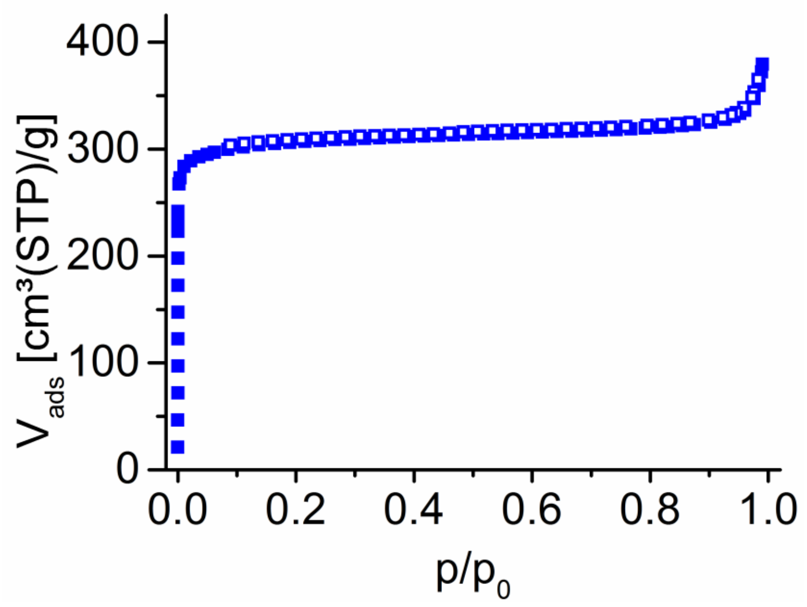

Supplementary Figure 3: Nitrogen sorption measurement of CAU-23.

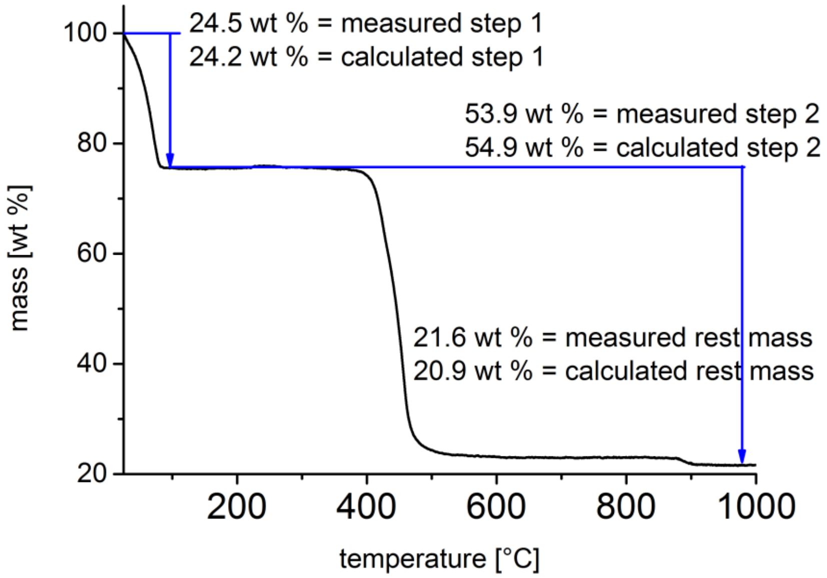

Supplementary Figure 4: Thermogravimetric measurements of CAU-23.

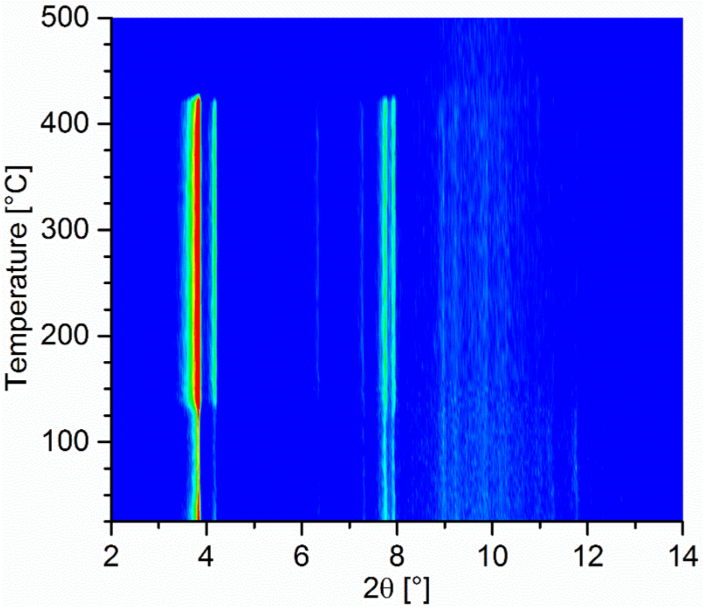

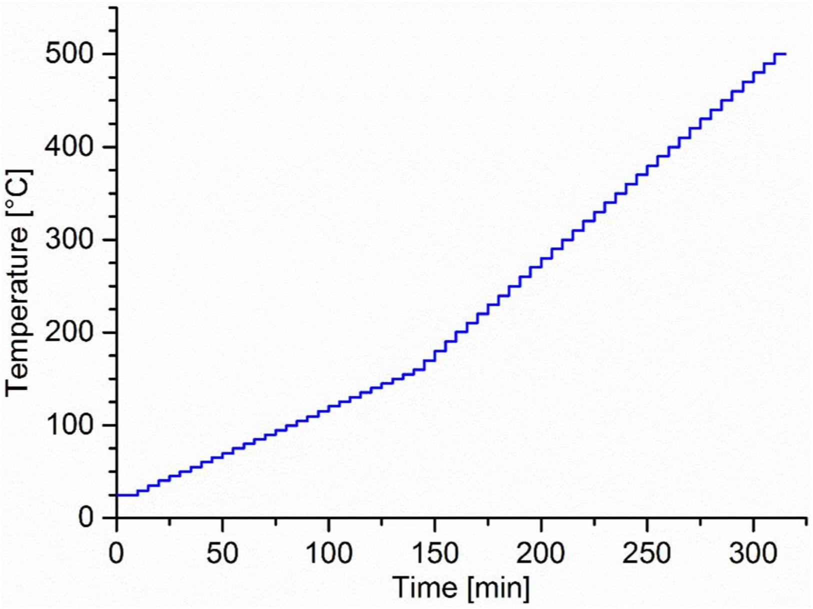

Supplementary Figure 5: (top) Temperature dependent PXRD measurements of CAU-23. (bottom) Temperature-time profile of temperature dependent PXRD measurements of CAU-23.

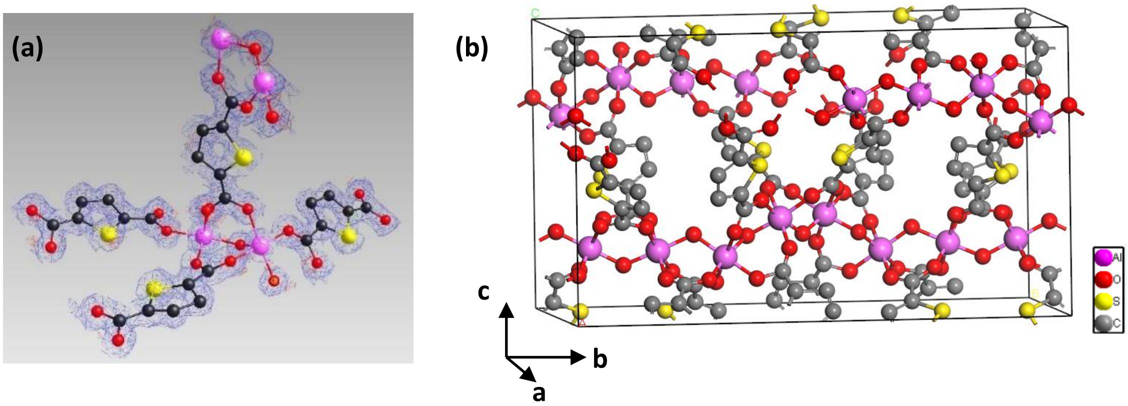

Supplementary Figure 6: a, Asymmetric structure unit combined with electron density map. The electron density map is represented by F_{obs} (blue, 1.2 Å^{-3}) and F_o-F_c (green (positive value at 0.63 Å^{-3}), red (negative value at -0.63 Å^{-3})). **b,** Atomic structure model in the full unit cell of CAU-23 obtained from electron diffraction data.

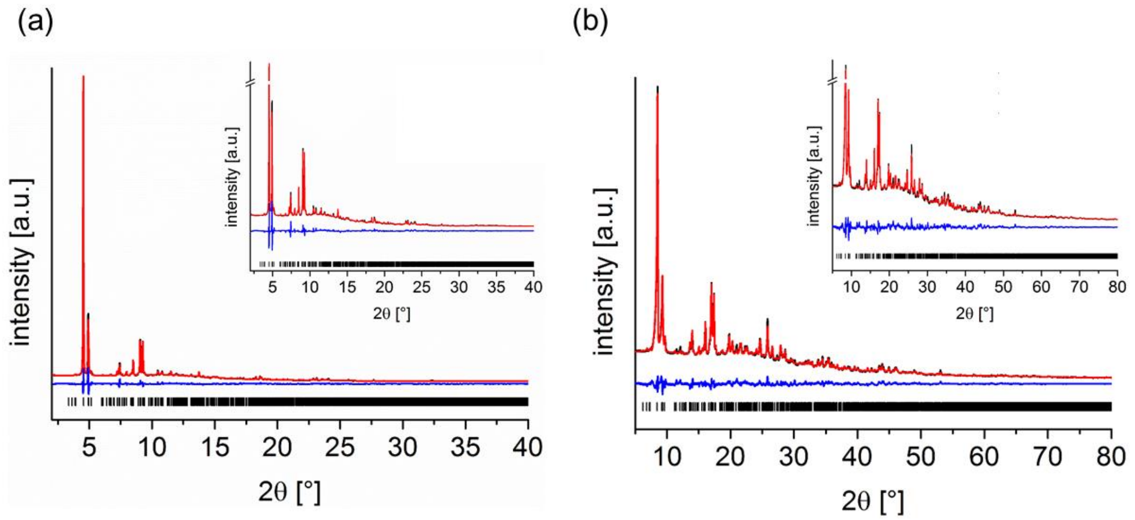

Supplementary Figure 7: Observed (black), calculated (red) and difference (blue) PXRD profiles and allowed reflections (black lines) for the Rietveld refinement of CAU-23 in dry (a) and wet state (b)**.**

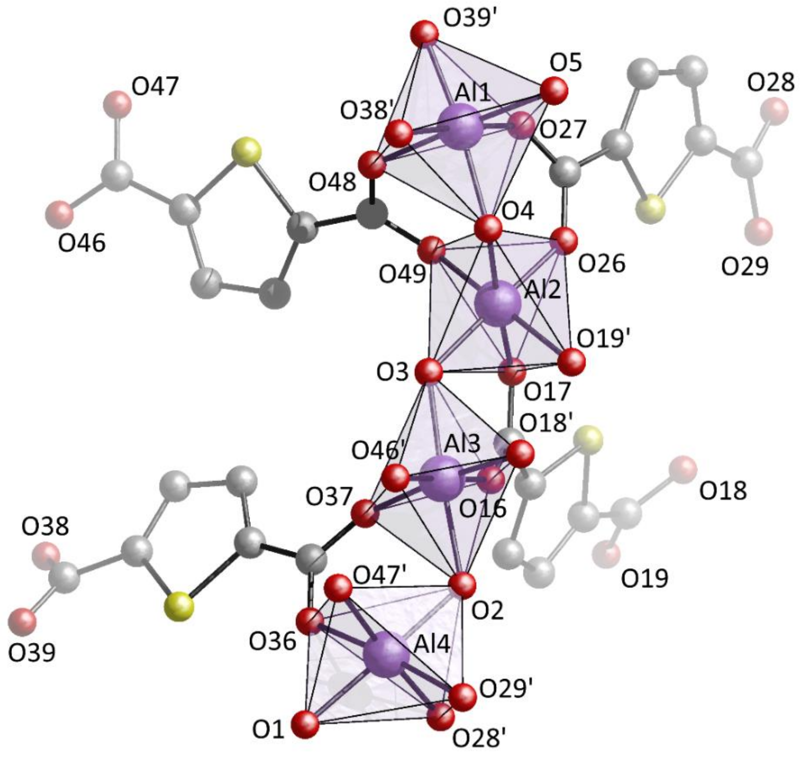

Supplementary Figure 8: Extended asymmetric unit of CAU-23-wet. For clarity, the coordination sphere of the metal site is completed by symmetry equivalent oxygen atoms, which are marked with a prime.

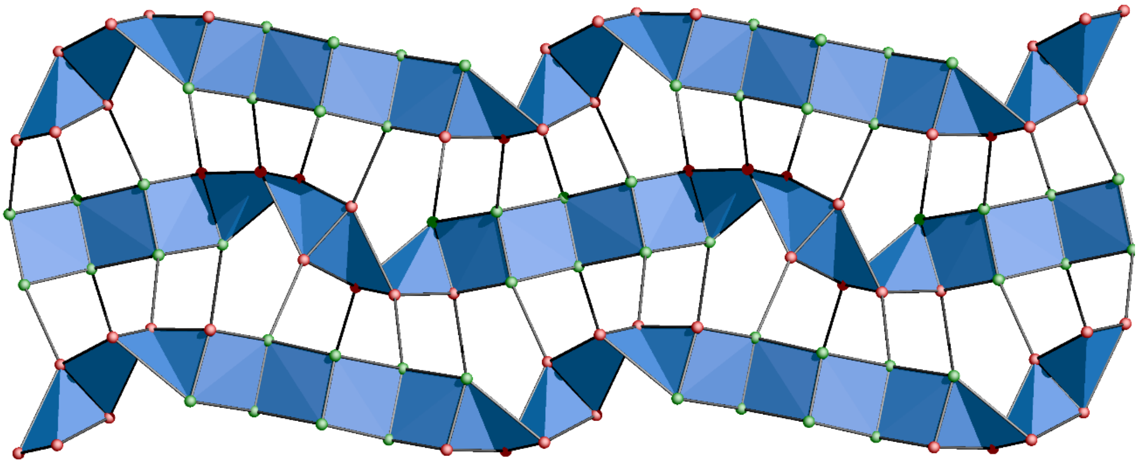

Supplementary Figure 9. Topological representation of the CAU-23 structure, viewed long [001]. Carboxylate carbon atoms (green and red nodes) are connected forming a 3-periodic net of CAU-23. The transitivity is 8 17, and the vertex symbol $\{4^2 6^2 8^2\}\{4^2 6^3 8\}$.

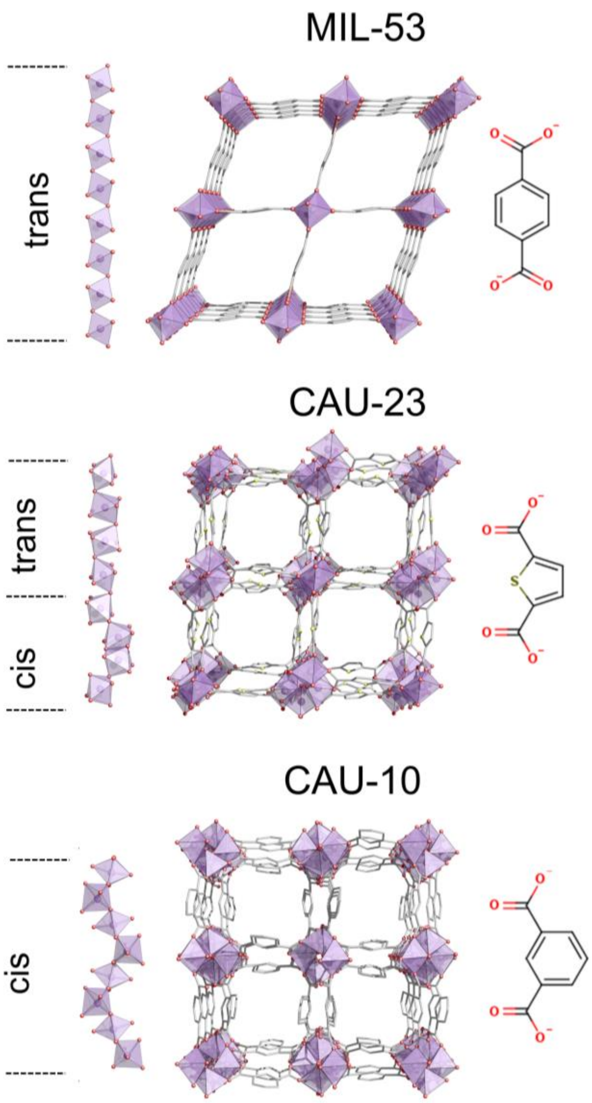

Supplementary Figure 10: Complete segment of the inorganic building unit (left), the structure (middle) and the incorporated linker molecules (right) in MIL-53-BDC,[4] CAU-23 (this work) and CAU-10.[5]

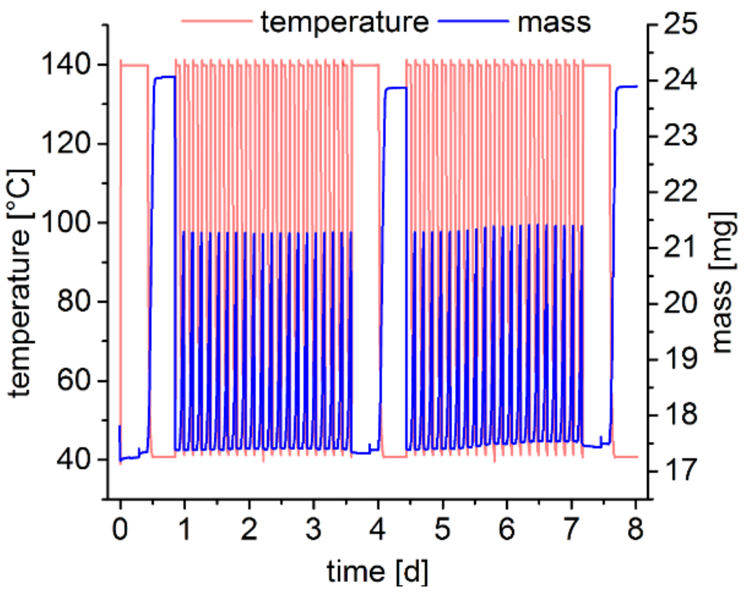

Supplementary Figure 11: Thermogravimetric adsorption/desorption cycling experiments for 40 cycles of CAU-23. Long segments at the beginning, in the middle and at the end of each experiment were conducted to determine the equilibrium loading of the sample.

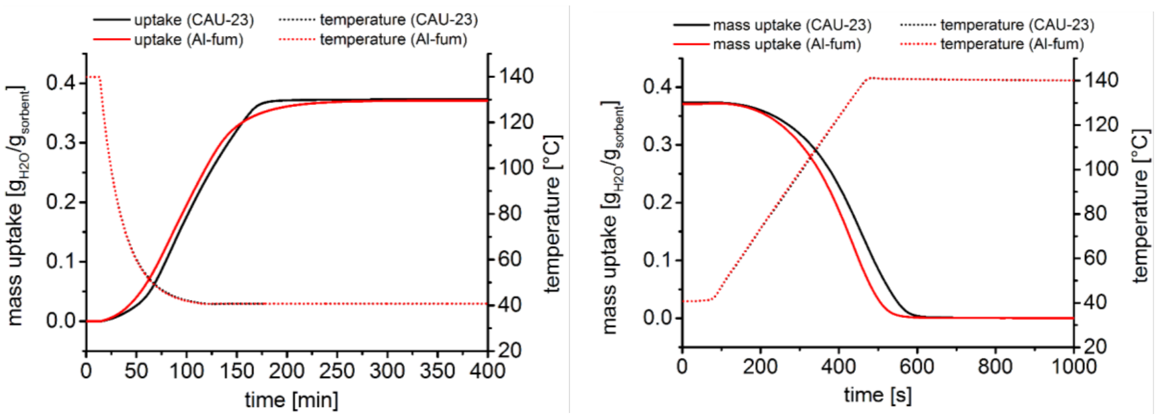

Supplementary Figure 12: Detail view of the thermogravimetric cycling experiment. Comparison of the adsorption (left) of the desorption (right) behavior of CAU-23 and Al-fum, using the equilibrium step after 20 cycles (t ~ 4 d and 4.5 d, respectively).

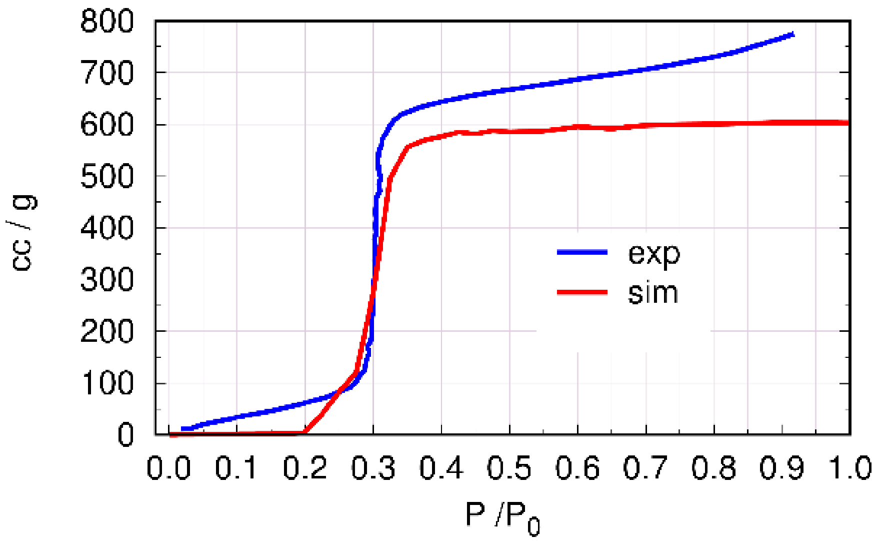

Supplementary Figure 13: Comparison between the water adsorption isotherms of CAU-23 derived from the experiment and the GCMC simulations at 25°C. The higher experimental uptake in comparison to the simulated one can be explained by the fact that CAU-23 is obtained in the form of very small crystallites. This leads to a large external surface as well as textural porosity, which in turn leads to higher observed uptakes.

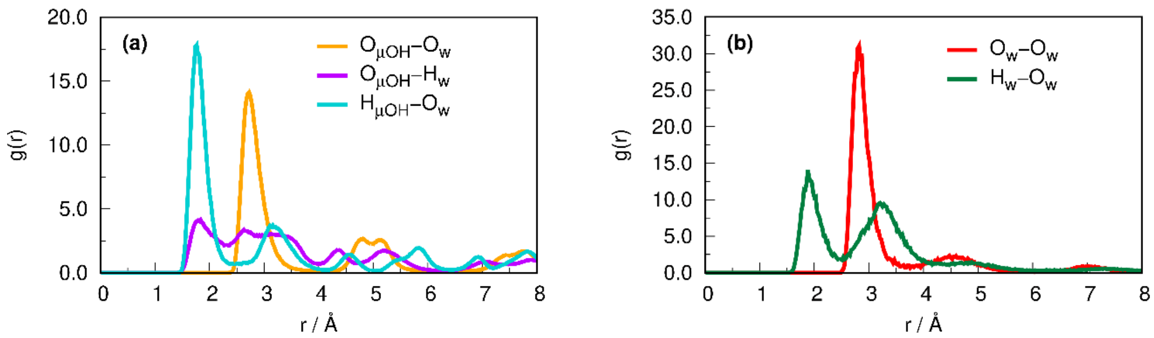

Supplementary Figure 14: Radial distribution functions of (a) intermolecular O···O and O···H pairs of water and μ-OH moieties on the pore wall of the CAU-23 structure, and (b) intermolecular $O_W \cdots O_W$ and $O_W \cdots H_W$ atomic pairs of water molecules calculated from the Monte Carlo simulations at low coverage.

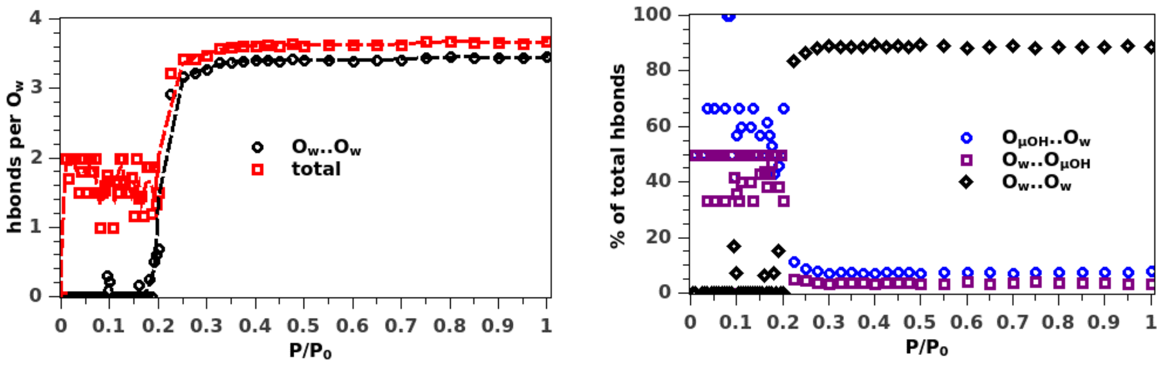

Supplementary Figure 15: Average number of hydrogen bond connections formed by adsorbed water molecules over the pressure range at T = 25 °C (left) and a detailed breakdown of different donor-acceptor scenarios of all the hydrogen bonds (right). Smoothed lines connecting real data points are drawn for guidance.

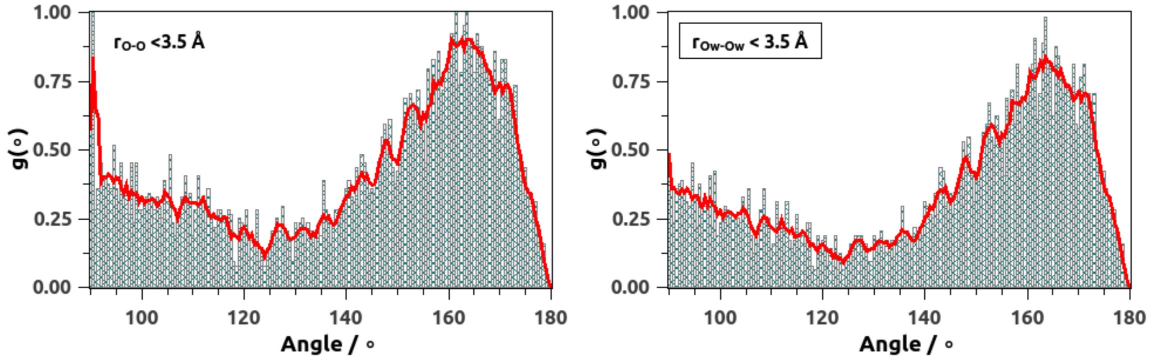

Supplementary Figure 16: Normalized probability distribution of donor-hydrogen-acceptor (D-H····A) angle formed by: **a,** adsorbed water molecules only, and also **b,** including the µ-OH moieties of the CAU-23 framework at saturation capacity.

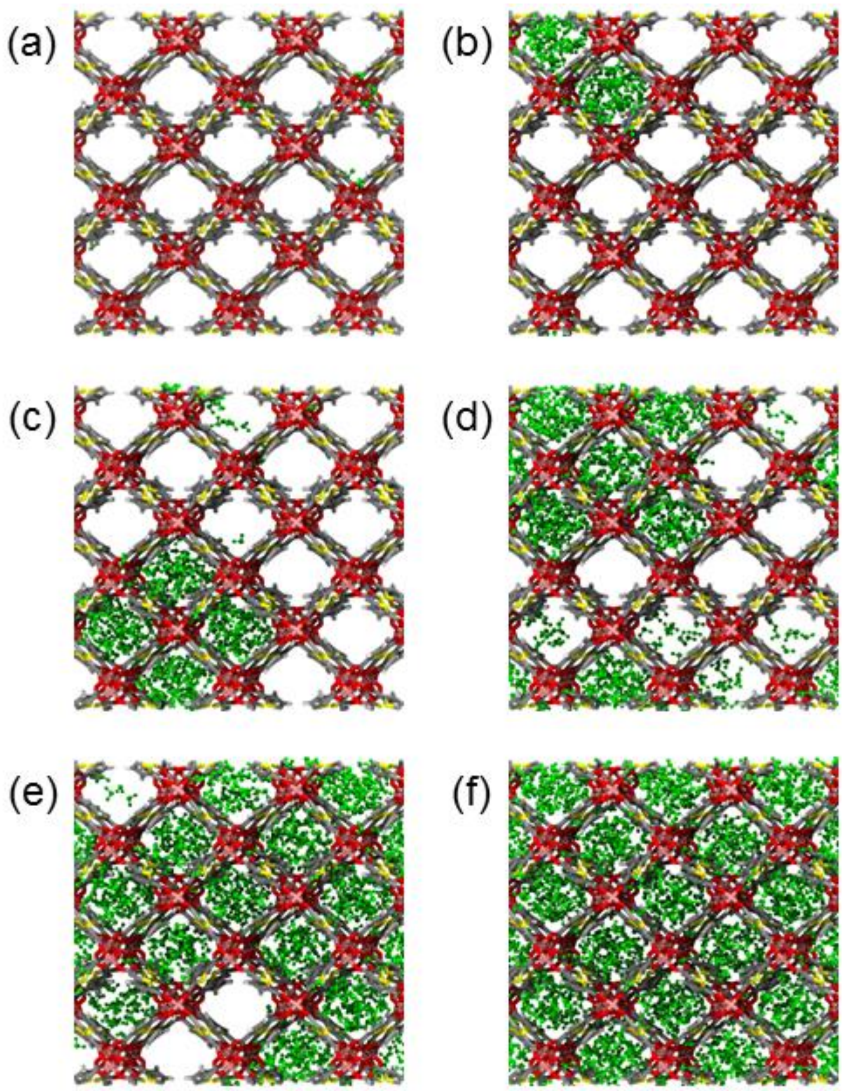

Supplementary Figure 17: Snapshots of the ensemble average of adsorbed water molecules within the 1D channel of CAU-23 at: (a) p/p_0 = 0.20, (b) p/p_0 = 0.225, (c) p/p_0 = 0.25, (d) p/p_0 = 0.275, (e) p/p_0 = 0.30, (f) p/p_0 = 0.35. For a distinctive depiction adsorbed water molecules are marked in green.

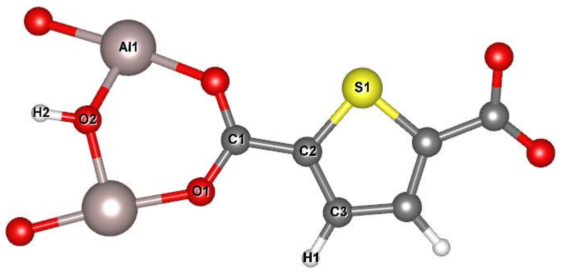

Supplementary Figure 18: A representative portion of the CAU-23 crystal structure showing the unique atom types in which the DFT derived RESP charges were assigned.

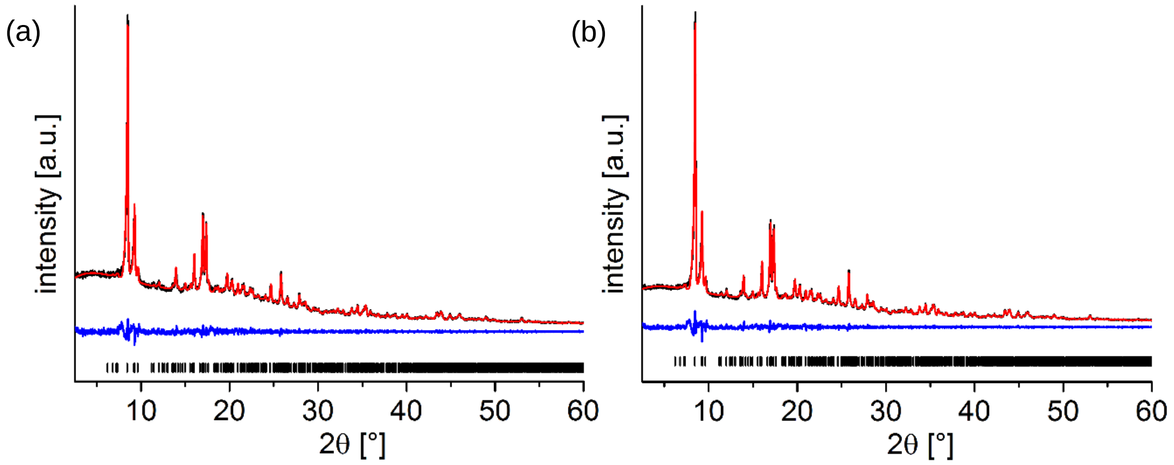

Supplementary Figure 19: Observed (dark), calculated (red) and difference (blue) PXRD profiles and allowed reflections (black lines) for the Le Bail fits of CAU-23 before (a) and after (b) a 5000 cycle stability test.

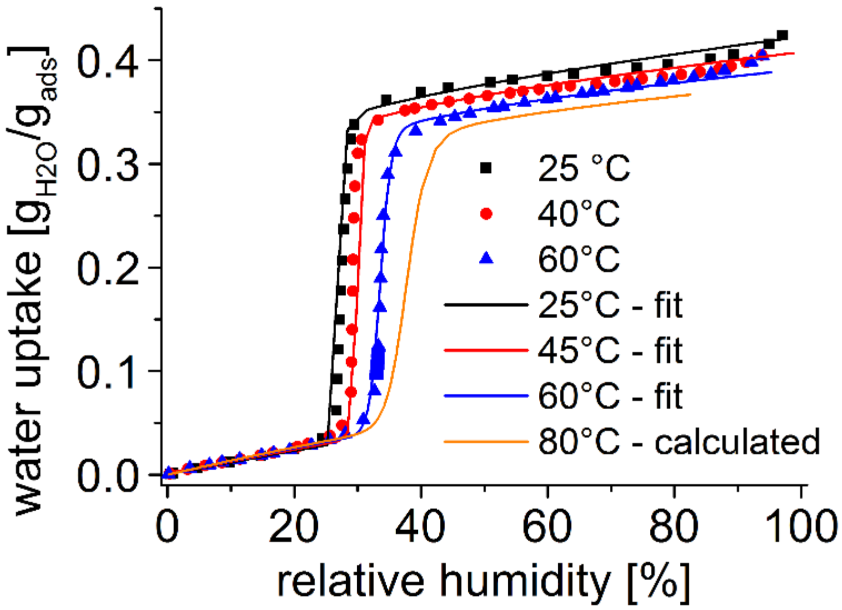

Supplementary Figure 20: Water adsorption isotherms at different temperatures and their fits or calculation of CAU-23.

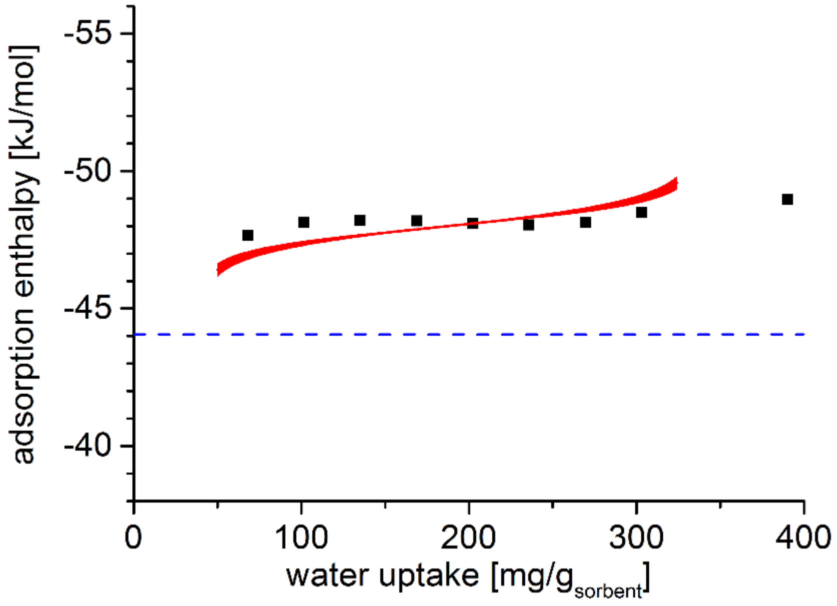

Supplementary Figure 21: Enthalpy determination and calculation for the steep increase of CAU-23. Blue dashed line is the condensation enthalpy of water.

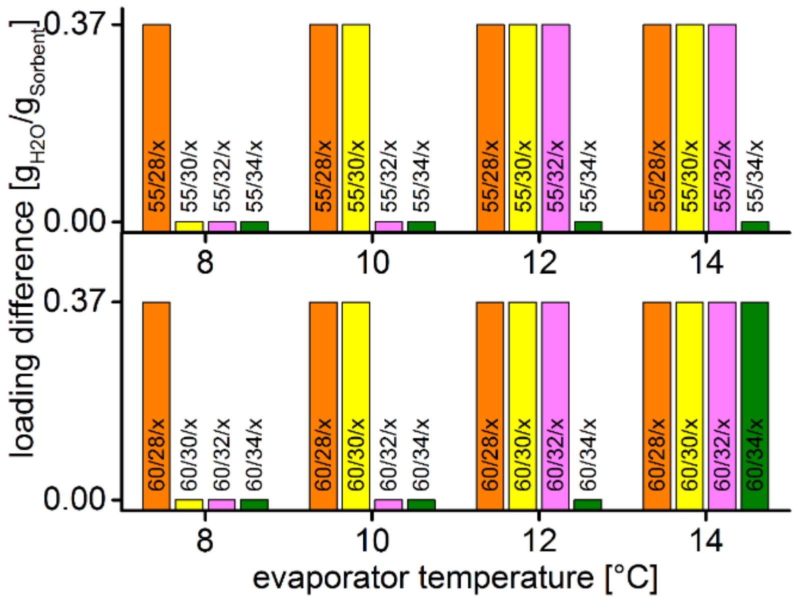

Supplementary Figure 22: Suitable temperature boundaries ($T_{driving}$/ $T_{condenser/heat\ rejection}$/ $T_{evaporator}$) with $T_{driving}$ = 55 (top) or 60 °C (bottom) extracted from Figures 5a and 5b. The values for $T_{condenser}$ vary from 28 to 34 °C and $T_{evaporator}$ from 8 to 14°C.

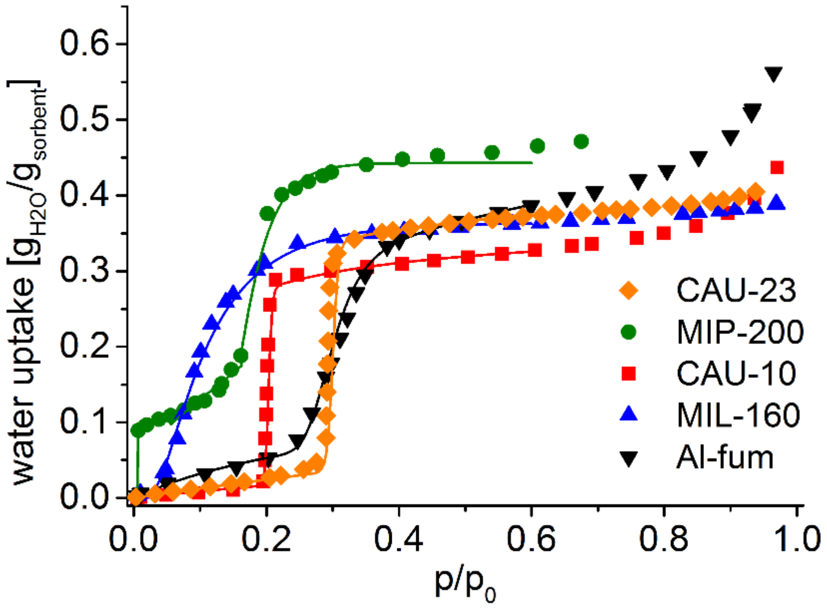

Supplementary Figure 23: Water adsorption isotherms for best-in-class adsorbents for adsorption driven chillers. Data source: CAU-23 (this work), Al-fum, MIL-160, CAU-10 (own measurements). For MIP-200 recently published data was used.[13] Solid lines represent the model fit.

Supplementary Table 1: Calculated COP values for a coated heat exchanger using different desorption temperatures.

m (adsorbent)	[kg]	1			
c_p (adsorbent)	[kJ/kg · K]	1.2			
m (heat exchanger)	[kg]	5			
c_p (heat exchanger)	[kJ/kg · K]	0.9			
Loading	[$g_{H2O}/g_{adsorbent}$]	0.35			
$T_{desired\ cold}$	[°C]	10			
$T_{back\ cooling/\ heat\ rejection}$	[°C]	30			
$T_{driving}$	[°C]	**60**	**70**	**80**	**90**
ΔT ($T_{driving}$ - $T_{heat\ rejection}$)	[°C]	30	40	50	60
Q_{evap}	[kJ]	858.9	858.9	858.9	858.9
Q_{des}*	[kJ]	937.2	937.2	937.2	937.2
Q_{IH}	[kJ]	36	48	60	72
$Q_{sens,\ heat\ exchanger}$	[kJ]	135	180	225	270
$\sum Q_{denominator}$	[kJ]	1108.2	1165.2	1222.2	1279.2
COP_C		**0.78**	**0.74**	**0.70**	**0.67**

*$\Delta H_{ads,CAU\text{-}23}$ = -48.2 KJ/mol

Supplementary Table 2: Elemental analysis of CAU-23.

	**wt% C**	**wt% H**	**wt% N**	**wt% S**
calculated	30.57	2.31	0	13.60
observed	31.89	3.04	0	13.94

Supplementary Table 3: cRED data processing and structure refinement details of CAU-23.

**Number of data sets**	4
**Wavelength** [Å]	0.0251
**Resolution** [Å]	1.13
**Crystal system**	Orthorhombic
**Space group**	$P2_12_12$ (No. 18)
**Unit cell *a*, *b*, *c*** [Å]	15.8(4),<br>24.1(5),<br>14.1(3)
**Total No. reflections**	19496
**Unique reflections (F > 4σ(F)/all)**	2038/3665
**Mean I/sigma(I)**	3.89
**Completeness**	0.959
**Parameters**	210
**R1 (F > 4σ(F))/R1(all)**	0.206/0.263
**GoF**	1.466

Supplementary Table 4: Crystallographic parameters of the structure refinement of CAU-23.

**State**	Dry	Wet
**Wavelength**	0.82531(1) Å	Cu-$K_{\alpha1}$
**Crystal system**	Orthorhombic	Orthorhombic
**Space group**	$P2_12_12$ (No. 18)	$P2_12_12$ (No. 18)
a [Å]	15.53(5)	15.4370(14)
b [Å]	24.20(8)	24.0600(16)
c [Å]	14.11(4)	14.2606(12)
**Volume** [Å^3]	5300(30)	5296.6(8)
R_{wp}(%)	4.981	4.145
R_{exp}(%)	0.342	0.338
**GoF**	14.533	12.270

Supplementary Table 5: Al-MOF with S-shaped H_2O isotherms. Employed linkers, -COOH opening angles and IBUs.[a]

Al-MOF	Linker	opening angle	IBU
MIL-160[6]	Furane dicarboxylic acid	116°	cis corner-sharing [AlO_6]polyhedra
CAU-10-H[5]	Isophthalic acid	120°	*cis* corner-sharing [AlO_6]polyhedra
CAU-10-PDC[6]	Pyridinedicarboxylic acid	120°	*cis* corner-sharing [AlO_6]polyhedra
CAU-23	Thiophenedicarboxylic acid	150°	[-4 *cis* - 4 *trans*-] corner-sharing [AlO_6]polyhedra
MIL-53-TDC[7]	Thiophenedicarboxylic acid	150°	*trans* corner-sharing [AlO_6]polyhedra
MIL-53-fum[8]	Fumaric acid	180°	*trans* corner-sharing [AlO_6]polyhedra

[a] Additionally, an Al-MOF containing pyrazoledicarboxylic acid (opening angle 150°) has been published recently, denoted MOF-303,[9] but in this publication only a structure model has been given, which nevertheless differs from the one observed in CAU-23.

Supplementary Table 6: LJ potential parameters and partial charges for the H_2O molecules taken from TIP4P/2005 model

**Atom type**	**σ** [Å]	**ε/k$_B$** [K]	**q (e$^-$)**
**O_e**	3.1589	93.200	0.0000
**H_e**	0.00	0.000	0.5564
**M_e**	0.00	0.000	-1.1128

Supplementary Table 7: LJ potential parameters and partial charges of the CAU-23 crystal structure

**Atom type**	**σ** [Å]	**ε/k$_B$** [K]	**q (e$^-$)**
**C1**	3.4730	47.857	0.677
**C2**	3.4730	47.857	-0.133
**C3**	3.4730	47.857	-0.121
**H1**	2.8644	0.000	0.142
**H2**	2.8644	0.000	0.382
**O1**	3.1180	30.193	-0.550
**O2**	3.1180	30.193	-0.864
**S1**	3.5948	0.000	0.100
**Al1**	4.0082	0.000	1.452

Supplementary Table 8: Results of the Le Bail fits of the coated CAU-23 samples before and after 5000 cycle stability test.

	**CAU-23 wet**	**CAU-23 before 5000 cycles**	**CAU-23 after 5000 cycles**
**space group**	$P2_12_12$	$P2_12_12$	$P2_12_12$
**a** [Å]	15.4370(14)	15.4718(16)	15.4874(12)
**b** [Å]	24.0600(16)	24.0760(14)	24.0836(13)
**c** [Å]	14.2606(12)	14.2349(15)	14.2280(11)
$\mathbf{R_{wp}}$	-	3.58	3.93
$\mathbf{R_{exp}}$	-	1.74	1.72
**Gof**	-	2.05	2.28

Supplementary Table 9: Gravimetric water uptake determination.

		**before cycle test**	**after cycle test**
**sample weight** [mg]	dry 1	33.2	33.9
	wet	44.3	45.4
	dry 2	33.0	34.0
**difference in loading** [wt%]	dry 1 - wet	33.4	33.5
	wet - dry 2	34.2	33.9
	average	33.8	33.7
**uptake taking binder mass into account** [wt%]		38.9	38.7

Supplementary Table 10: Volumetric water uptake, calculated from the crystal structure of the different metal-organic frameworks discussed for their usage in adsorption driven chiller systems.

	CAU-10[5,11]	CAU-23*	Al-Fum[8,12]	MIP-200[13]	MIL-160[6,14,15]
sum formula $_{network}$	$[Al(OH)(C_8O_4H_4)]$	$[Al(OH)(C_6SO_4H_2)]$	$[Al(OH)(C_4O_4H_2)]$	$[Zr_6O_4(OH)_4(C_{17}O_8H_8)_2(HCOO)_4]$	$[Al(OH)(C_6O_5H_2)]$
$z_{unit\ cell}$	16	16	4	3	16
M [g/mol]	208.11	214.13	158.05	1539.91	198.07
$m_{unit\ cell}$ [$g \cdot 10^{-21}$]	5.53	5.68	1.05	7.67	5.26
$V_{unit\ cell}$ [$Å^3$]	4781	5300	990	6475	4728
Density [g/cm^3]	1.16	1.07	1.06	1.18	1.11
usable water capacity [$g_{H2O}/g_{sorbent}$]	0.30	0.37	0.35	0.30	0.36
volumetric water uptake [$g_{H2O}/cm^3_{sorbent}$]	0.35	0.40	0.37	0.36	0.40

*This work

Supplementary Methods

Elemental analysis

The samples were analyzed on an Elementar Vario Micro Cube elemental analyzer (CHNS). The sum formula of the calculated values for CAU-23 is $[Al(C_6H_2O_4S)(OH)] \cdot 1.2\ H_2O$ (water content is varying with the external relative humidity and temperature; Supplementary Table 2).

IR spectroscopy

The infrared spectra of the reaction products were measured on a Bruker Alpha-P IR spectrometer. For the drying of the compound it was heated to 100 °C for 16 h (Supplementary Figure 2).

Nitrogen sorption measurement

The nitrogen sorption experiment was carried out at a BEL Japan Inc. Belsorpmax at 77 K. The sample was activated at 150 °C for 16 h under reduced pressure (< 0.1 mbar).

The nitrogen sorption measurement of CAU-23 shows a type I isotherm (Supplementary Figure 3). The BET area is determined as S_{BET} = 1250 m²/g (16 points < p/p_0 = 0.02) and the micro pore volume as V_{mic} = 0.48 cm³ / g (at p/p_0 = 0.5). Using the program Materials Studio 5.5 (BIOVIA, formerly Accelrys), the theoretical accessible surface area of CAU-23-dry was calculated as S_{BET} = 1330 m² / g. Hydrogen atoms of the thiophene ring were inserted in ideal positions for theoretical surface calculations and the kinetic radius of nitrogen was set to 1.8 Å.

Thermogravimetric measurements

The thermogravimetric curve was recorded in air on a Linseis STA PT 1600 (heating rate = 4 K min^{-1}, gas flow = 20 mL/min). For the evaluation the formula $[Al(OH)(C_6H_2O_4S)] \cdot 3.8\ H_2O$ was used (water content is varying with the external rh and temperature). Since the decomposition product at 1000 °C is not crystalline the composition Al_2O_2S was used (Supplementary Figure 4).

Temperature dependent PXRD measurements

Temperature dependent powder X-ray diffraction patterns were recorded on a Stoe Stadi P Combi diffractometer in transmission geometry equipped with Mo-$K_{\alpha1}$ radiation, a curved germanium monochromator and a linear MYTHEN detector with an aperture angle of 17° using a furnace (Supplementary Figure 5).

Structure determination details from electron diffraction data with cRED of CAU-23

The structure of CAU-23 was solved by direct methods using SHELXT, and all the atom positions were found directly (Supplementary Figure 6). The structure was refined against the cRED data using SHELXL.[3] Atomic scattering factors for electrons and isotropic atomic displacement parameters were used for the refinement without adding any restraints. The structure converged rapidly with chemically reasonable bond lengths and angles. The selected crystallographic data are given in Supplementary Table 3, and the structure details are provided in the supporting cif file.

Structure refinement details of CAU-23 using PXRD data

Due to the extremely high intensities typical for synchrotron based PXRD measurements or in-house measurements with long expose time (38 h), R_{exp} is very low, resulting in a GoF that is higher than the typical value for in-house data (Supplementary Figures 7-8, Supplementary Table 3).

Stability test of coated CAU-23

The Le Bail fits were performed using TOPAS Academic 6 (Supplementary Table 8).[10]

The water uptake capacity of coated CAU-23 was investigated gravimetrically. The sample was exposed to different temperatures, 100 °C for 2 h, 16 h with 71.1 % relative humidity at 20.1 °C realized by saturated aqueous sodium chloride solution (150 g NaCl in 100 mL water) and 100 °C for 2 h again. The mass of every sample was measured and the difference was calculated and used to determine the uptake (Supplementaey Table 9).

Supplementary Notes

1 Benefits for low adsorption temperature on a adsorption driven chiller system

There are three important factors why a low desorption temperature has multiple benefits compared to finding MOFs with high adsorption capacities (ideally high capacities and low desorption would be the ultimate goal).[1]

First, by lowering the driving temperature other applications may come into range for adsorption cooling that yet cannot be used efficiently (district heating, geothermal heating, data centers. …). For example our main effort in the past was to achieve High Performance Cooling using the waste heat from CPUs or GPUs in order to drive the process to cool the boundaries. Although new CPUs may withstand temperatures up to 90°C, throttling is activated for temperatures above 70°C.

Second, whereas the cooling energy per cycle is directly correlated to the water uptake, the regeneration energy is directly correlated to the heat of adsorption and the temperature needed to remove the water molecules. In a real device, all components like the heat exchanger, binder and pipes have to be heated up additionally to the necessary driving temperature to remove the adsorbed water. This can be summed up as sensible heat Q_{sens} of these parts and is consumed in every cycle (Q_{IH} = isosteric heating of the MOF). This additional term is added to the COP_C (coefficient of performance for cooling) calculation, thus lowering the COP.

$$Q_{sens} = \mathrm{Q}_{sens,HX} + Q_{sens,binder} + Q_{sens,pipes} = \sum m\, c_p \Delta T \qquad (1)$$

$$COP_\mathrm{C} = \frac{Q_\mathrm{evap}}{Q_\mathrm{des} + Q_\mathrm{IH} + \boldsymbol{Q_{sens}}} \qquad (2)$$

The higher the necessary driving temperature the more energy is lost by the needed sensible heat in every single cycle. Taking this point into account, we have carried out an estimation on the importance of a reduced desorption temperature on the COP for a coated heat exchanger (Supplementary Table 1), keeping the other parameters constant.

The reduction of the desorption temperature from 90 to 60 °C leads to a significant enhancement of the COP from 0.67 to 0.78, an improvement of 15 %. This calculation demonstrates the change for only one cycle, but the consumed sensible heat has to be multiplied by the amount of cycles performed during lifetime of the device (>100 000). Thus it leads to considerable overall amounts, showing the immense influence of the driving temperature.

Taking the higher necessary desorption temperature for Al-fum of 90 °C into account, whereas its adsorption enthalpy is comparable to CAU-23 ($\Delta H_{evap,Al\text{-}fum}$ ~ -50 kJ/mol,[2] $\Delta H_{evap,CAU\text{-}23}$ = -48.2 KJ/mol, this work), the sensible heat Q_{sens} will have a much larger negative impact on the COP of Al-fum than it is for CAU-23 ($T_{desorption}$ = 60°C).

Third, under working conditions a low driving temperature (here < 60 °C) is also advantageous even if higher driving temperatures are available (e.g. waster heat). For a real system containing a MOF-coated heat exchanger (device) thermodynamics comes into play and the heat flow from the source to the fluid to the heat exchanger to the MOF (heat resistance chain) must take place to desorb the water molecules. In order to have a relevant heat transfer a temperature difference is needed. This is described in Fourier´s law in its integral form as

$$\dot{\boldsymbol{Q}} = \lambda \cdot \mathrm{A} \frac{T_1 - T_2}{\mathrm{d}} \qquad (3)$$

with $\dot{Q}$= heat flow rate, λ = thermal conductivity, A = cross sectional area, $T1$, $T2$ = temperatures on the different sides and d = thickness of the wall. This equation can also be given in the differential form.

$$q = -k\nabla T \qquad (4)$$

with q = heat flux density, k = conductivity, ∇T = temperature gradient

Reaching the minimum desorption temperature, the MOF starts to desorb efficiently while consuming heat (desorption is endothermic). Thus a larger heat flux into the material leads to

a faster desorption. A higher temperature difference ($T1$-$T2$ or ∇T) will lead to a higher heat flux and consequently a shorter desorption cycle can be achieved. Thus more cycles per time are possible, which directly leads to higher cooling power output of the system.

2 Topological description of CAU-23

Carboxylate carbon atoms have been chosen as the nodes of the underlying 3-periodic net (Aupplementary Figure 6). There are 8 unique nodes and 17 unique edges (transitivity 8 17) in this net. All 8 unique nodes are 4-connected, forming a helical twist along the *b*-axis. Four (green) of these nodes are located on the straight segments, while the other four (red) form a full helical twist. Around each green node/vertex are two quadrilaterals, two hexagons, and two octagons; around each red vertex are two quadrilaterals, three hexagons, and one octagon. Consequently, the vertex symbol of this net is $\{4^2 6^2 8^2\}\{4^2 6^3 8\}$. All red nodes at the helical segments have the same vertex condition of $\{4^2 6^3 8\}$, while the green nodes at the straight segment have the vertex condition $\{4^2 6^2 8^2\}$.

3 Comparison of the water uptake of CAU-23 and Al-fum

To get an insight into the water sorption kinetics of CAU-23 and to be able to compare it to another state of the art material (Al-fum)[2], cycling thermogravimetric measurements were carried employing the same experimental set up and parameters (T_{min}, T_{max}, relative humidity, gas flow rate, similar sample weight). The results are shown in Supplementary Figure 11.

Both samples, Al-fum and CAU-23 show a very similar adsorption/desorption behavior under the cycling conditions. The desorption behavior of CAU-23, following the equilibrium step after 20 cycles (t ~ 4 d), was compared to those available for Al-fum (Supplementary Figure 12). Both materials show a similar desorption speed, small differences are due to a slightly larger dry weight of CAU-23 (17.3 mg) than for Al-fum (15.3 mg). This shows, that the sorption kinetics of CAU-23 will be appropriate for its use in ADCs.

Both materials show similar desorption and adsorption properties. The small differences are due to different dry weight of CAU-23 and Al-fum (17.3 and 15.3 mg, respectively) and the shape of the H_2O adsorption isotherm of Al-fum.

4 Structural trends in selected Al-MOFs with S-shaped water sorption isotherms

To establish possible trends for the future choice of linker molecules that could lead to suitable Al-MOFs for ADC applications, a comparison of known Al-MOFs with S-shaped water sorption isotherms was carried out.

The influence of the opening angle between the two –COOH groups of the linker on the MOF structures was systematically studied. This investigation led to the discovery of CAU-10-H. A linear linker as fumaric acid led to a MIL-53-type structure (MIL-53-fum). Decreasing the angle between the –COOH groups to 120° led to different inorganic building units and different crystal structures exhibiting different water sorption properties (Supplementary Table 5). Within similar opening angles (116-120°) using pyridinedicarboxylic acid and furandicarboxylic acid instead of isophthalic acid the CAU-10 structure is observed. The incorporation of a heteroatom (oxygen or nitrogen) leads to an increase in the hydrophilicity of the framework and H_2O uptake at lower relative humidity values. Increasing the opening angle to 150° seems to lead to no predictable correlation between the linker and the resulting MOF structure. Thus with thiophendedicarboxylic acid (opening angle 150°) the MIL-53 type structure and CAU-23 with a new inorganic building unit are observed. Both show very interesting but different water sorption properties.

5 Volumetric uptake capacity of MOFs with S-shaped water sorption isotherms

The volumetric uptake capacity of a coating is an important number for the construction of a heat exchanger. The volumetric uptake capacity of the crystals can be directly calculated from the crystal structure (Supplementary Table 10). All MOFs that have been discussed for ADC applications exhibit very similar uptake capacities, varying from 0.35 to 0.40 g/cm^3.

The uptake capacity of a coated heat exchanger depends on the composition of the MOF/binder composite. Hence a CAU-23/binder sample was prepared according to the procedure given for the coating of a complete heat exchanger with CAU-10.[11] Thus a CAU-23/binder (SilRes® MP50E by Wacker Chemistry) composite was coated on cleaned aluminum metal sheets (4 x 4 cm coating size, 16.7 wt% binder, 83.3 wt% dry MOF). The coating thickness, as well as the mass of the coatings, were measured and thus the composite density was determined to be 0.46 g/cm^3 (each measurement was repeated five times and the values were averaged). Taking the binder mass and the gravimetric water uptake capacity for CAU-23 into account, a volumetric uptake capacity of 0.14 g_{H2O}/cm^3 composite is obtained. These values are highly depending on the way the coating is manufactured, as changing the binder content or the binder itself will change the density of the coating. To provide an estimate value for a full scale heat exchanger, a heat exchanger with 1 L volume and 0.9 m^2 surface is considered with a coating thickness of 300 µm. This results in a volumetric density of 37.8 $g_{H2O}/L_{heat\ exchanger}$.

Supplementary References

1. Barth, H.-J. & Scholl, S. eds. *Innovative Heat Exchangers* (Springer International Publishing, Cham, 2018).
2. Jeremias, F., Fröhlich, D., Janiak, C. & Henninger, S. K. Advancement of sorption-based heat transformation by a metal coating of highly-stable, hydrophilic aluminium fumarate MOF. *RSC Adv* **4,** 24073–24082; 10.1039/C4RA03794D (2014).
3. Sheldrick, G. M. A short history of \it SHELX. *Acta Crystallogr. .A* **64,** 112–122; 10.1107/S0108767307043930 (2008).
4. Loiseau, T. *et al.* A Rationale for the Large Breathing of the Porous Aluminum Terephthalate (MIL-53) Upon Hydration. *Chem.– Eur. J.* **10,** 1373–1382; 10.1002/chem.200305413.
5. Fröhlich, D. *et al.* Water adsorption behaviour of CAU-10-H. A thorough investigation of its structure-property relationships. *J. Mater. Chem. A* **4,** 11859–11869; 10.1039/C6TA01757F (2016).
6. Wahiduzzaman, M., Lenzen, D., Maurin, G., Stock, N. & Wharmby, M. T. Rietveld Refinement of MIL-160 and Its Structural Flexibility Upon H2O and N2 Adsorption. *Eur. J. Inorg. Chem.* **2018,** 3626–3632; 10.1002/ejic.201800323 (2018).
7. Tschense, C. B. L. *et al.* New Group 13 MIL-53 Derivates based on 2,5-Thiophenedicarboxylic Acid. *Z. Anorg. Allg. Chem.* **643,** 1600–1608; 10.1002/zaac.201700260 (2017).
8. Alvarez, E. *et al.* The Structure of the Aluminum Fumarate Metal–Organic Framework A520. *Angewandte Chemie* **127,** 3735–3739; 10.1002/ange.201410459 (2015).
9. Fathieh, F. *et al.* Practical water production from desert air. *Sci. Adv.* **4**; 10.1126/sciadv.aat3198 (2018).
10. Coelho, A. A. TOPAS and TOPAS-Academic: An optimization program integrating computer algebra and crystallographic objects written in C++. *J.Appl. Crystallogr.* **51,** 210–218; 10.1107/S1600576718000183 (2018).

11. Lenzen, D. *et al.* Scalable Green Synthesis and Full-Scale Test of the Metal–Organic Framework CAU-10-H for Use in Adsorption-Driven Chillers. *Adv. Mater.* **30,** 1705869; 10.1002/adma.201705869 (2018).
12. Kummer, H. *et al.* A Functional Full-Scale Heat Exchanger Coated with Aluminum Fumarate Metal–Organic Framework for Adsorption Heat Transformation. *Ind. Eng. Chem. Res.* **56,** 8393–8398; 10.1021/acs.iecr.7b00106 (2017).
13. Wang, S. *et al.* A robust large-pore zirconium carboxylate metal–organic framework for energy-efficient water-sorption-driven refrigeration. *Nat. Energ.* **3,** 985–993; 10.1038/s41560-018-0261-6 (2018).
14. Cadiau, A. *et al.* Design of hydrophilic metal organic framework water adsorbents for heat reallocation. *Adv. Mater.* **27,** 4775–4780; 10.1002/adma.201502418 (2015).
15. Permyakova, A. *et al.* Synthesis Optimization, Shaping, and Heat Reallocation Evaluation of the Hydrophilic Metal-Organic Framework MIL-160(Al). *ChemSusChem* **10,** 1419–1426; 10.1002/cssc.201700164 (2017).
