# Supplementary material for: A metal–organic framework for efficient water-based ultra-low-temperature-driven cooling
Source: Nat Commun. 2019 Jul 9;10:3025. doi: 10.1038/s41467-019-10960-0 (PMC6616384; doi:10.1038/s41467-019-10960-0)
Supplement: Supplementary file 2 — Transparent Peer Review File [file 41467_2019_10960_MOESM2_ESM.pdf]

**Editorial Note:** This manuscript has been previously reviewed at another journal that is not operating a transparent peer review scheme. This document only contains reviewer comments and rebuttal letters for versions considered at *Nature Communications*. Mentions of the other journal have been redacted.

REVIEWERS' COMMENTS:

Reviewer #1 (Remarks to the Author):

The authors well addressed the comments raised by the reviewers and carefully revised the manuscript. I am completely satisfied with the revised manuscript and in my opinion, this is very good piece of work and definitely acceptable to Nature Communications.

Reviewer #2 (Remarks to the Author):

I have carefully reviewed this article in the past while it was submitted to [**redacted**]. I made a few relatively minor comments, and in this submission to Nature Communications, I think all of my comments have been satisfactorily addressed. Considering the novelty and significance of this presented study, I can now recommend publication. I have no further comments.
